# Four simple rules that are sufficient to generate the mammalian blastocyst

**DOI:** 10.1371/journal.pbio.2000737

**Published:** 2017-07-12

**Authors:** Silas Boye Nissen, Marta Perera, Javier Martin Gonzalez, Sophie M. Morgani, Mogens H. Jensen, Kim Sneppen, Joshua M. Brickman, Ala Trusina

**Affiliations:** 1 StemPhys, Niels Bohr Institute, University of Copenhagen, Copenhagen, Denmark; 2 The Danish Stem Cell Centre, DanStem, University of Copenhagen, Copenhagen, Denmark; 3 Transgenic Core Facility, University of Copenhagen, Copenhagen, Denmark; 4 CMOL, Niels Bohr Institute, University of Copenhagen, Copenhagen, Denmark; University of Pennsylvania, United States of America

## Abstract

Early mammalian development is both highly regulative and self-organizing. It involves the interplay of cell position, predetermined gene regulatory networks, and environmental interactions to generate the physical arrangement of the blastocyst with precise timing. However, this process occurs in the absence of maternal information and in the presence of transcriptional stochasticity. How does the preimplantation embryo ensure robust, reproducible development in this context? It utilizes a versatile toolbox that includes complex intracellular networks coupled to cell—cell communication, segregation by differential adhesion, and apoptosis. Here, we ask whether a minimal set of developmental rules based on this toolbox is sufficient for successful blastocyst development, and to what extent these rules can explain mutant and experimental phenotypes. We implemented experimentally reported mechanisms for polarity, cell—cell signaling, adhesion, and apoptosis as a set of developmental rules in an agent-based in silico model of physically interacting cells. We find that this model quantitatively reproduces specific mutant phenotypes and provides an explanation for the emergence of heterogeneity without requiring any initial transcriptional variation. It also suggests that a fixed time point for the cells’ competence of fibroblast growth factor (FGF)/extracellular signal—regulated kinase (ERK) sets an embryonic clock that enables certain scaling phenomena, a concept that we evaluate quantitatively by manipulating embryos in vitro. Based on these observations, we conclude that the minimal set of rules enables the embryo to experiment with stochastic gene expression and could provide the robustness necessary for the evolutionary diversification of the preimplantation gene regulatory network.

## Author summary

The first 4.5 days of mammalian embryo development proceeds without maternal information and is remarkably robust to perturbations. For example, if an early embryo is cut in half, it produces 2 perfectly patterned, smaller embryos. Where does the information guiding this development come from? Here, we explore this issue and ask whether a model composed of a simple set of rules governing cell behavior and cell—cell interactions produces in silico embryos. This agent-based computational model demonstrates that 4 rules, in which a cell makes decisions based on its neighbors to adopt polarity, make lineage choices, alter its adhesion, or die, can recapitulate blastocyst development in silico. By manipulating these rules, we could also recapitulate specific phenotypes at similar frequencies to those observed in vivo. One interesting prediction of our model is that the duration of cell—cell communication through fibroblast growth factor (FGF) signaling controls scaling of a region of the blastocyst, and we confirmed this experimentally. Taken together, our model specifies a set of rules that provide a framework for self-organization, and it is this self-organizing embryogenesis that may be an enabler of stochastic variation in evolution.

## Introduction

**Fig 1:**
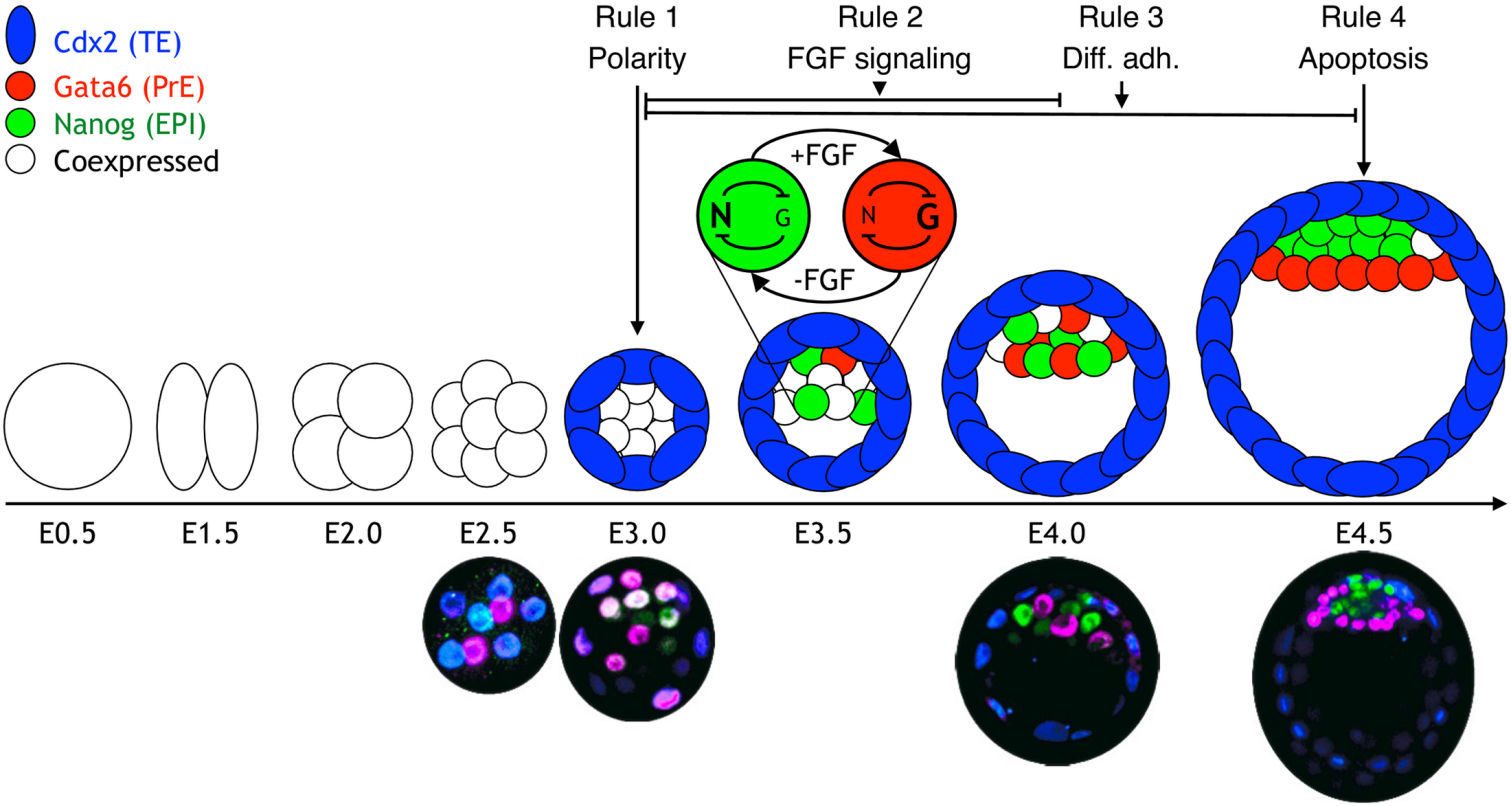
Schematic of the early embryonic development. The zygote (embryonic day [E] 0.5) undergoes 3 rounds of cleavage divisions, resulting in 8 cells at E2.5. During the next round of division, the blastomeres undergo compaction and become polarized, resulting in the outer trophectoderm (TE) (blue) and the inner cell mass (ICM) (still coexpressing Gata6 and Nanog). The TE expresses the transcription factor caudal-related homeobox 2 (Cdx2). At E3.5, a cavity has formed, and the ICM is positioned at 1 side of the embryo. At this stage, the ICM transcription factors, Gata6 (red) and Nanog (green), are expressed in a mutually exclusive salt-and-pepper pattern in some cells. At E4.5, Nanog- and Gata6-expressing cells have physically segregated into 2 distinct layers and are developmentally restricted to either the epiblast (EPI) or primitive endoderm (PrE). The lower panel shows immunostaining of embryos at different stages during preimplantation development. Color coding is the same as in the panel above. The timing of the 4 different rules that we apply is indicated on top of the diagram.

Early mammalian development is a fascinating example of how deterministic spatiotemporal patterns emerge at the level of cell populations from highly stochastic regulatory components. During mouse preimplantation development, 2 sequential lineage decisions take place [1] (Fig 1), and these decisions are marked by the expression of lineage-determining transcription factors. The first decision happens between embryonic day (E) 2.5 and 3.0, as the morula is formed. The outer cells of the embryo express the transcription factor caudal-related homeobox 2 (Cdx2) and form the trophectoderm (TE), while the inside cells express sex-determining region Y-box 2 (Sox2) [2] and form the inner cell mass (ICM). The morula then cavitates, forming the blastocyst, and the ICM differentiates into 2 lineages: Gata6-expressing cells form the primitive endoderm (PrE), an epithelial layer adjacent to the blastocoel cavity, and Nanog-expressing cells form the epiblast (EPI) enclosed by the TE and the PrE. The specification of EPI and PrE is a gradual process that involves the initial specification of cell types in a salt-and-pepper distribution throughout the ICM and then their progressive segregation by E4.5, the time of implantation [3,4]. All future lineages of the embryo, including the germ line, are generated from the EPI. The TE and PrE lineages will produce the support structures required for placental and yolk sac development.

These early decisions are remarkable in that they proceed in the apparent absence of maternal information, and that the cells undergoing these differentiation decisions remain competent for respecification up to around E3.5. Either the removal of blastomeres or the aggregation of multiple morulae as late as E3.5 can produce developmentally competent embryos, albeit at a lower success rate [5,6]. In fact, single blastomeres from 32-cell stage mouse blastocysts can generate entire mice [7,8].

While the analysis of mutant phenotypes has suggested the broad outlines of several regulatory mechanisms involved in embryo development, the robust and regulative nature of early patterning cannot currently be explained. Based on the literature, we have identified 4 major regulatory themes:

### (1) Segregation of TE and ICM at the 16-cell stage and emergence of apico-basal polarity in TE

At compaction, E2.5–E3.0, the outer cells of the embryo become polarized, express the transcription factor Cdx2, and differentiate into TE. At E3.0, the apical membrane of outer cells expresses several proteins that have a known role in cell polarity [9–13]. The acquisition of polarity starts with compaction at the 8-cell stage, in which apical domain is developed at the contact-free surface. The apical domain is inherited asymmetrically at the next cell division and was shown to play an important role in defining inner and outer cells through cells sorting due to differential contractility [14]. How the TE fate becomes limited to only outer cells is not fully understood, but it is suggested to be a combined effect of contractility, Hippo, and Notch pathways. The Hippo signaling pathway is normally activated at high cell densities and, in this context, is specifically activated in the inner cells [15–18] in which it induces phosphorylation and degradation of the transcriptional coactivator Yes-associated protein 1 (Yap). In outer cells with higher contractility, the levels of phosphorylated Yap are higher [14]. In the absence of Hippo activation, the TEA domain family member 4 (Tead4) binds Yap and cooperates with Notch signaling to induce the transcription factor Cdx2 and specify the TE [19]. In addition, tight junctions are formed between the TE cells in a plane perpendicular to the polarity axis [20,21], and this may further reinforce TE polarity.

### (2) Signaling via FGF/ERK regulates the specification of ICM cells to PrE or EPI in a salt-and-pepper pattern

At E3.0, the inside cells down-regulate Cdx2 but express octamer-binding transcription factor 4 (Oct4) and become ICM. The cells of the ICM initially express both Gata6 and Nanog, but early variations in expression are thought to be propagated by the production of fibroblast growth factor (FGF) 4 downstream of Nanog and higher levels of the FGF receptor (FGFR2) in cells expressing higher levels of Gata6. EPI precursors expressing Nanog secrete FGF4, promoting a PrE fate in neighboring cells [22–26]. Consistently, ex vivo manipulation of the FGF pathway from E2.5 to E4.0 can change the fate of ICM cells [27–30]. It has been shown in vitro that Nanog and Gata6 repress each other intracellularly [31–36]. Moreover, FGF/extracellular signal—regulated kinase (ERK) signaling enhances Gata6 expression while repressing Nanog [36–39]. Finally, modulating the FGF4 level is sufficient to convert all ICM cells to either PrE (high FGF4) or EPI (low FGF4) [40,41].

### (3) Differential adhesion contributes to PrE and EPI lineage segregation, with PrE lining the surface of the EPI and adjacent to the blastocoel cavity

During the period that cells are making a lineage decision between EPI and PrE, cell movement occurs within the ICM [42]. Chazaud et al. [3] showed that initially EPI and PrE progenitors arise in a heterogeneous mosaic pattern and later physically segregate into the appropriate cell layers, which are finally separated by a basal lamina. It was proposed that the cell movements contribute to cell sorting and may be due to differential adherence of progenitor cell types, which has been observed in vitro [43,44]. There have also been several reports on differences in the expression level of the adhesion molecule integrin β1 receptor during PrE differentiation in vitro between the 2 ICM lineages [45–47]. Several other mechanisms contribute to the formation of the “layered” pattern [48], including down-regulation of transcriptional programs in inappropriately positioned cells or apoptosis [4].

### (4) ICM cells undergo apoptosis at the time of PrE specification

As the blastocyst expands, the ratios of the PrE and EPI are self-regulating, as paracrine interactions control proliferation and apoptosis. In particular, the cytokine Leukemia inhibitory factor (LIF) appears to regulate the relative size of the PrE and EPI. LIF is secreted by TE cells, and the corresponding receptor complex is found in the ICM [49]. LIF has been shown to act both on EPI and PrE fate. It blocks maturation in the EPI, and it supports proliferation and cell survival in the PrE [50,51]. In addition, atypical protein kinase C (aPKC) and platelet-derived growth factor (PDGF) signaling promote survival of PrE precursors that reach the surface of the ICM [52,53]. Furthermore, a considerable number of ICM cells undergo apoptosis around the time of PrE formation [4,54,55]. Plusa et al. [4] showed that there is a steady increase in the rate of apoptosis from E4.0–E4.5. They reported that PrE precursors are more likely to undergo apoptosis when they are deep within the ICM than when they are positioned along the cavity lining.

Here, we hypothesized that a combination of these 4 themes could together explain the robust nature of blastocyst formation. We have conceptualized and unified these themes as rules in a rule-based model to investigate their relative contribution to the robustness of early embryo development.

Both the initial specification of the TE and the ICM and differentiation and segregation of PrE and EPI have been modeled in silico at different levels. Chickarmane et al. [56] focused on intracellular transcription networks generating the 3 stable states (EPI, PrE, and TE). Bessonnard et al. [57] modeled 25 static ICM cells on a grid and addressed how cell—cell communication via the FGF/ERK pathway establishes the right proportion of EPI and PrE cells. Krupinski et al. [58,59] modeled the mechanical interaction of cells, focusing on the role of polarity in Cdx2 partitioning, as well as differential adhesion and directed movements for the segregation of PrE, EPI, and TE into 3 distinct layers. In these models, the growth of the blastocyst is driven by the growing cavity, and all cells have similar apolar interactions, albeit of different strength. None of these models address the role of polarity in TE cell—cell interaction, apoptosis, or aspects of the emergence of blastocyst scaling [60–62].

The existing in silico models provide important insight into the individual mechanisms driving cell specification during preimplantation development but do not provide a unified framework of early embryo development as a self-organizing system [63]. Recently, such a framework, using rule-based modeling, has been proposed for the specification of synaptic partner cells [64]. Here, we use a similar approach to propose a minimum set of rules to quantitatively model early blastocyst development. Our aim is not to recapitulate the precise timing of mouse development, but to show that with a simple set of rules we could capture blastocyst patterning. As evolution can produce changes in the timing and wiring of the gene regulatory network, the patterning of the mammalian embryo should be able to tolerate stochasticity; our aim is to show that the 4 simple rules can enable this robustness.

We focus on 4 rules that include polarity, cell—cell communication via FGF4 signaling, differential adhesion, and apoptosis. Using a series of in silico 2D simulations, we quantify the relative contribution of these 4 elements to early embryonic development. To facilitate comparison to published genetic studies, we have validated this approach in 3D simulations. By introducing polar interactions between TE cells, we show that the development (including cavity formation) is self-organized and does not require an a priori assumption of the growing cavity. Moreover, based on these 4 rules, we found that we could effectively simulate experimental embryo manipulation: our model successfully reproduces a range of experimentally observed mutant phenotypes and predicts that the time point of FGF activation could be a clock that dictates the relative size of EPI and PrE in scaling experiments. Consistent with the notion that the timing and duration of FGF/ERK activation is the essential variable in proportioning these 2 lineages, we found that delaying ERK activation by 24 hours resulted in a quantitative reduction in PrE specification.

## Results

### Approach

**Fig 2:**
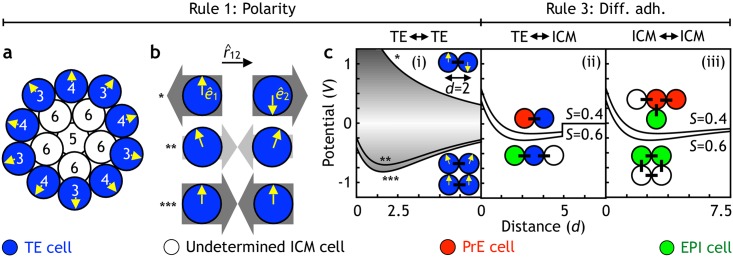
The overview of the physical interactions between cells. **(a)** Polarity is applied to the surface cells at the 16-cell stage. Cells with 4 or fewer neighbors (numbers) acquire polarity (arrows) pointing radially out from the center of the embryo. These cells become the trophectoderm (TE) (blue), while the inner cells with 5 or more neighbors become the undetermined inner cell mass (ICM), coexpressing primitive endoderm (PrE) and epiblast (EPI) markers (white). **(b)** The 3 types of interaction between 2 TE cells (blue). In case the polarity unit vectors (arrows, *ê*) are or become antiparallel (*), the repulsion is maximal (dark gray arrows); although, in our simulations, this configuration does not occur. When the polarities are misaligned, the potential is smaller (**), and the attraction is weak (light gray arrows). Finally, when the 2 polarities are parallel, the potential is strong (***), and the cell attraction is strongest. **(c)** The potentials applied between the different cell types in the model. All cells are illustrated as unit circles, and the potentials are given by Eq 2. (i) Potential between TE cells depends on the orientation of cell polarities (arrows) given by Eq 1. To implement nearest-neighbor interaction potential, TE cells only interact with other TE cells within *d* < 2.5. (ii) Interaction potential between the TE and ICM cells (PrE cells in red, EPI cells in green, and undetermined ICM cells in white). The range of the potential is limited to about 2 cell diameters (*V* = 0 for *d* > 5). PrE progenitors interact weaker with TE cells (*S* = 0.4) compared to either undetermined ICM or EPI cells (*S* = 0.6). (iii) Interaction potentials between ICM cells. As in (ii), PrE progenitors interact weaker with any of the ICM cells (*S* = 0.4) compared to EPI—EPI, EPI—undetermined ICM, or undetermined ICM—undetermined ICM (*S* = 0.6).

In a growing blastocyst, cells are tightly packed and adhere to each other. Similarly to earlier in silico models [58,65], we simulate this by introducing an interaction potential in which cells repel each other at a distance smaller than their typical size and attract at longer distances (see Fig 2 and Materials and methods for details of the potential). The interactions between all cell types are the same, except in 2 cases. First, to simulate differential adhesion, the attraction is set to be weaker with and among PrE cells. Second, in contrast to previous models, the physical forces between TE cells are assumed to depend on cell polarity such that 2 TE cells adhere to each other when their polarity is pointing in the same direction, and cells are positioned next to each other in the plane perpendicular to the polarity axis. Biologically, this would correspond to a well-known phenomenon of tight junctions forming in a plane perpendicular to the polarity axis [66].

The modeled blastocyst grows as cells divide. Cell division is simulated by selecting a cell at random and introducing a daughter cell between the mother and its nearest neighbor. In case of TE, 1 daughter cell inherits polarity including the orientation of the polarity from the mother cell.

To conceptually capture the 4 major themes outlined in the Introduction, we have formulated the following 4 rules:

### Rule 1: Develop polarity, if surface cell

At E3.0, we define the outer cells by counting the number of nearest neighbors (shown in Fig 2a). Cells with fewer than 5 nearest neighbors are assigned TE fate and polarity, pointing radially outwards from the center of the cell mass. We do not aim at recapitulating how the polarity is established and how inner and outer cells are defined in a real embryo. Instead, we focus on the role of polarity in outer cells after it has been established. To take into account that TE cells are about twice as big as the rest of the cells, each TE cell is simulated by 2 unit circles. Polarity of the TE cells is assumed to lead to polar interactions that can be thought of as tight junctions forming in the plane perpendicular to the TE polarity. We simulate this by a polarity-dependent attraction factor, *S* (see Eq 1), that is maximal for 2 neighboring cells if their polarity is oriented in the same direction and perpendicular to the position vector (illustrated in Fig 2b). At every time point, the strength of attraction, *S*, between 2 neighbor TE cells is given by:

$$S=-1.4\;\left({\hat{e}}_{1}\;\times \;{\hat{r}}_{12}\right)\cdot \left({\hat{e}}_{2}\;\times \;{\hat{r}}_{21}\right)$$ (1)

Where ê is the polarity unit vector of a TE cell, and $\hat{r}$ is the unit distance vector between 2 neighbor TE cells. The prefactor of 1.4 assures that TE cells are tightly packed but nonoverlapping. Notice that this polar interaction favors the formation of a single-layer sheet with cells positioned perpendicular to the polarity axis and disfavors compact structures (Fig 2b).

The dynamics of the polarity vectors is governed by simple damped dynamic equations as in Eq 3 (see Materials and methods). Proliferation of TE was reported to be about twice as fast compared to ICM [67], so we set the rate of TE cell division to be 2-fold that of ICM cells. In all lineages, daughter cells inherit the mother’s fate and polarity.

### Rule 2: Switch fate, if surrounded by too many cells of the same type (FGF4 signaling)

From E3.0 to E4.0, the FGF signaling pathway becomes important for lineage segregation in the ICM. Bessonnard et al. [57] suggests that the FGF/ERK pathway coupled with the intracellular mutual inhibition between Nanog and Gata6 act together to ensure the fidelity of initial EPI and PrE specification. The simplified logic behind this process can be reduced to the intracellular inhibition and extracellular activation between Nanog and Gata6 (shown in the network in Fig 1 at E3.5):

1. Intracellular mutual repression between Nanog and Gata6 assures that cells are in either PrE or EPI progenitor states.
2. Extracellular Nanog > Gata6 activation represents the fact that Nanog-expressing cells (EPI) secrete FGF4, which, at high enough concentrations, can induce Gata6 in a neighboring cell. We assume FGF4 effects are short-ranged, limited to 1 cell diameter [68].
3. Extracellular Gata6 > Nanog activation represents an implicit effect of Gata6 (PrE) cells lowering extracellular FGF in their neighborhood (both due to receptor/ligand internalization and lack of FGF4 secretion) and thus promoting Nanog in neighbor cells.

The mutual inhibition between Nanog and Gata6 is, in effect, an intracellular positive feedback loop. When reduced to 1 variable (e.g., Nanog), the network reveals a combination of intracellular amplifying positive feedback with extracellular inhibition of Nanog in neighboring cells. This representation suggests a Turing mechanism that results in both local amplification and global inhibition (see S2 Fig). Simulations based on this Turing mechanism predict that ICMs would maintain the ratio of PrE (or EPI)/ICM, irrespective of embryo size.

At E3.0, all ICM cells are in an “undetermined state” coexpressing low levels of both Gata6 and Nanog (Fig 1), and the cell specification process is started as all these cells begin to express FGF4 [40,69]. This initial step follows the same simplified logic outlined above, as long as we assume that once specified, Gata6 cells have a lower concentration of FGF4 in their neighborhood than undetermined ICM cells.

In the model, we implement this logic by monitoring the number of nearest-neighbor cells with high FGF4 (EPI or undetermined ICM) versus low FGF4 (PrE) (as in Fig 2a). At cell division, the likelihood of a mother and a daughter cell to convert to PrE is proportional to the fraction of high FGF4 (EPI or undetermined ICM) cells in the neighborhood ($P\left(PrE\right)=\frac{\#\;of\;high\;FGF4\;neighbors}{\#\;of\;ICM\;neighbors}$), and, conversely, the likelihood to convert to EPI is *P*(*EPI*) = 1 − *P*(*PrE*). As a result of this simple rule, in our simulations, cells undergo the specification into salt-and-pepper pattern of Nanog/Gata6 cells from the initial state of unspecified ICM. In addition, all cells can potentially convert their identity between PrE and EPI later (E3.5 to E4.0), as the blastocyst grows. Although the identity of cells can be modulated in ex vivo blastocyst culture in response to FGF treatment or inhibition [27], once cell identity is established, conversion is quite rare in unmanipulated culture conditions [70]. In our simulations, the rate of conversion is low and asymmetric, which is in line with observations by Xenopoulos et al. [70].

### Rule 3: Less adhesion, if PrE progenitor

Differential adhesion is activated once the cells specify their identity (at E3.5). It is implemented by a single change in the attraction factor for PrE precursors from *S* = 0.6 to *S* = 0.4. Biologically, this corresponds to lower adhesive properties of PrE cells. Thus, the attraction factor between 2 EPI, 2 TE cells, or an EPI cell and an ICM or TE cell remains at *S* = 0.6, while the attraction factor, *S*, between 2 PrE or PrE and any other cell type is reduced to *S* = 0.4. These potentials are shown in Fig 2c. TE cells only interact with their nearest neighbors (i). Limiting the range of the TE—ICM potential (ii) to about 2 cell diameters allows the model to capture the symmetry breaking event (at E3.5), with ICM and cavity forming at the opposite sides. ICM cells are assumed to interact with all the other ICM cells (e.g., by protrusions), which is implemented by a global potential without explicit cutoffs (iii). As a result, PrE precursors migrate away from the EPI core and form the PrE layer at the surface of the ICM, facing the cavity. With this rule, the model predicts that if the TE were removed at the blastocyst stage, in the isolated ICM, the EPI would end up surrounded by PrE (S1 Fig and S3 Movie). This is consistent with the experimental observation that in the cluster of mixed EPI/PrE cells, PrE cells migrate to the outer layer surrounding the EPI core [43].

### Rule 4: Die, if in a wrong position (LIF-induced apoptosis)

At E4.5, PrE precursors in the EPI core, i.e., cells surrounded by more than 3 non-PrE precursors, undergo apoptosis to ensure that failures in lineage segregation are not incorporated into EPI development.

To compare with experimental results from 3D blastocysts, we used simple scaling relationships, converting between 2D and 3D (see Materials and methods). We have also validated our approach in 3D simulations (S2 Movie). All the major steps were the same as in 2D, with 1 modification: In 2D, TE cells would always have 2 nearest TE neighbors. We identify these 2 TE cells as nearest neighbors if they are within a certain distance. However, in 3D, this approach fails as one may obtain cell centers within a cell diameter that are not nearest but next-nearest neighbors. To account for this and to find the list of “true” nearest neighbors, we have developed a method that separates nearest from next-nearest neighbors. We evaluate if a potential nearest neighbor is closest to the given cell—and thus included as its true nearest neighbor—or if it is closer to another cell in the neighborhood and thus not counted as nearest neighbor (see Materials and methods for details). While this neighborhood assignment is necessary for the stability of the TE in the 3D model, it is not sufficient, as without polarity the TE cells would collapse into a clump, and cavity cannot be formed.

**Fig 3:**
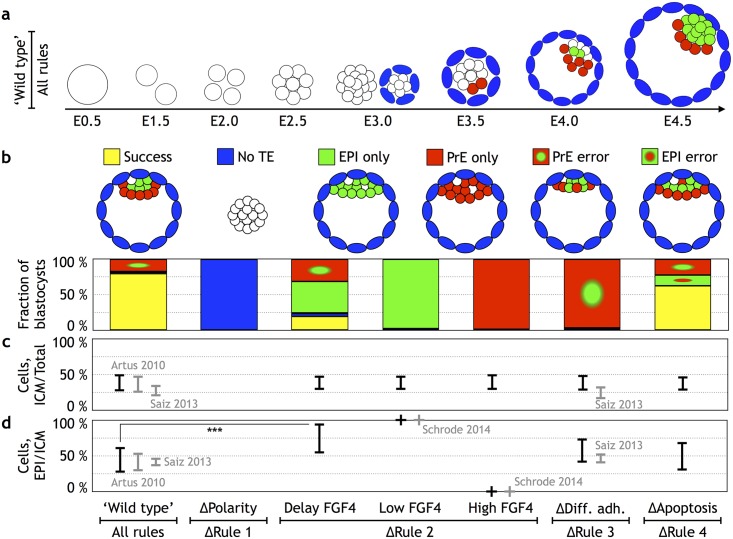
Quantifying the relative contribution of the rules to the robustness of the blastocyst development. **(a)** Screenshots from a representative simulation (compare with Fig 1, and see also S1 Movie). At embryonic day (E)3.0, both the configuration before and after adding polarities are shown. Undetermined inner cell mass (ICM) cells are white, trophectoderm (TE) cells are blue, epiblast (EPI) cells are green, and primitive endoderm (PrE) cells are red. **(b)** Quantification of the developmental success in in silico blastocysts at E4.5. The configuration of the successfully developed blastocyst is shown in the upper-left panel. The occurrence of such a configuration (79% out of 200 simulations in wild type) is color coded by yellow. Perturbing 1 of the 4 rules results in 5 additional configurations (upper panel): no TE formation (blue), EPI only (green), PrE only (red), EPI progenitor within the PrE (red with a green dot), and PrE progenitors found within the EPI (green with a red dot). The “ΔRule 2 low/high FGF” represents cases in which fibroblast growth factor (FGF) signaling is depleted or is in excess FGF4, which corresponds to data in Yamanaka et al. [27] and Saiz et al. [71]. The “ΔRule 2 delay FGF4” represents the case in which FGF signaling was inhibited for 24 hours from E2.5–E3.5, and the inhibitor is removed at E3.5–E4.5. **(c)** The fraction of ICM cells to the total number of cells at E4.5 in the 7 different types of embryos. **(d)** The fraction of EPI cells to ICM cells at E4.5. Both in (c) and in (d), experimentally reported numbers are presented in gray, while black error-bars represent numbers predicted by the model. The simulation data can be found in S1 Data. To compare 2D simulations with 3D experimental results, the numbers were rescaled to 3D (see Materials and methods). The simulation results are similar in 3D model (see S2 Movie).

In order to quantify the importance of each of the rules, we specified the “successful” configuration of the blastocyst at E4.5 to be the one in which (i) TE cells are segregated from ICM cells and form a shell; (ii) the cavity is formed, and the ICM is positioned at one side of the cavity; (iii) ICM cells segregate in 2 distinct layers with the PrE positioned between the cavity and the EPI cells; (iv) and no isolated EPI cells are in the PrE layer nor isolated PrE in the EPI. By comparing the outcome of our simulations to the criteria above, we quantified the fraction (out of 200 simulations for each condition) of “successful” in silico blastocysts (Fig 3b). Representative screenshots from a successful simulation are shown in Fig 3a and in S1 Movie.

### “Wild type”

We found that with all 4 rules in place, the success rate is high (79%, Fig 3b, see also S2 Movie), suggesting that these rules together are sufficient for development of blastocysts up to E4.5. We also challenged these 4 rules in 3D simulations and found that they were sufficient to generate 3D blastocysts (S2 Movie). For the sake of simplicity, we will compare the impact of specific perturbations in these rules using 2D simulations. In 2D simulations, the fraction of ICM/total cells was 39 ± 12%, and the EPI/ICM fraction was 44 ± 18%, both of which are in a good agreement with experimental data [48,51,53] (Fig 3c and 3d). Of the 21% failure in our simulations, about 20% occurred when—as a result of stochastic update—the fraction of ICM cells (ICM/total cell number) was low. As a result, there were not enough cells available to close the PrE layer, resulting in a PrE error. In about 1% of the cases, the TE broke, either due to failure of maintaining contacts between the surrounding TE cells or right after polarities have been added at E3.0. This occurs if the embryo is in a “tight configuration” in which adding another ICM cell disrupts the shell of TE cells. This error, we believe, is attributed to our choice of potential and noise parameters and might happen even more rarely if the parameters are fine-tuned.

In successful cases, embryos transited through a salt-and-pepper pattern, eventually separating PrE from EPI. To what extent this pattern is salt-and-pepper, i.e., how big are the regions with the same cell types, depends on several factors. The longer the range of FGF4 signal, the larger are the patches of the same cells; on the other hand, the size of the patches is also increased if the differential adhesion molecules are expressed at the same time as cell specify (as is assumed in our model). While visually we do observe patches of different sizes in published data, validation of this aspect of our model will require single-cell quantification of 3D imaging.

### ΔRule 1

To quantify the role of polar interaction, we “switched off” the polarity by setting the attraction factor for all cells to be the same as for undetermined ICM cells (*S* = 0.6). Without polarity (ΔPolarity case) all cells clustered together; consequently, there was no cavitation and no characteristic shell-like layer of TE cells forming (Fig 3b). These results agree well with the observations in mouse mutants and knockdowns targeting polarity pathways: homozygous mutation in downstream regulator of Yap/Taz signaling, *Tead4* [60,62]; chemical inhibition of RHO-ROCK signaling (required for apical-basal polarity), knockdown of Pard6b (a component of PAR-aPKC) by RNA interference (RNAi), disturbing the apical complex aPKC/PAR6 by small interfering RNA (siRNA), downregulating aPKC/PAR3 by injecting double-stranded RNA (dsRNA), or Prickle2 mutants [72–76]—all result in severe polarity defects (including the absence of tight junctions), and all fail to form blastocoel.

### ΔRule 2

Elimination of the second rule can be carried out by modulating the FGF concentration either up or down. As expected, low FGF concentration (−FGF in Fig 1 and S3 Movie) in our model resulted in no PrE formation and an ICM consisting of only EPI at E4.5. These cells were found in a clump consisting of several layers in one side of the blastocyst. This spatial configuration of EPI cells is in agreement with the observed *FGF4*- and *FGFr2*- mutants [40,77–80].

Also as expected, the maintenance of a constant FGF/ERK on state (+FGF in Fig 1 and S4 Movie) resulted in ICMs composed solely of PrE, consistent with the experimental results from introducing an excess amount of FGF [27,40,41] (Fig 3d). As a result of stronger adhesion between EPI cells compared to adhesion between PrE cells, the ICM cells clump more in the “EPI only” (low FGF) case compared to the “PrE only” (high FGF) case.

The clumping of the “PrE only” cells is in disagreement with the experimental observation by Yamanaka et al. [27] in which PrE cells are positioned on one side of the blastocyst in 1 layer lining the TE. This disagreement is likely because in our model, the difference between PrE and EPI cells is limited to differences in adhesive properties and does not include the reported apical-basal polarity of the PrE cells [18]. Adding polar interactions to the PrE layer in our model will disfavor “clumping” and make PrE cells line along the TE layer. While polarity of the PrE may add to the robustness of the blastocyst patterning, we chose not to include it into the current model as, within the criteria for success we specified, it does not seem to be necessary for the successful development of the “wild-type blastocyst.”

### ΔRule 3

Noticeably, the failure rate is close to 100% when the differential adhesion between the Gata6 and Nanog positive cells is neutralized (by setting *S* = 0.5 for all the ICM cells) (Fig 3b, ΔDifferential adhesion case, see also S5 Movie). At E4.5, PrE progenitors remained distributed in a salt-and-pepper pattern; consequently, a considerably higher fraction of the PrE progenitors underwent apoptosis. The ICM/total cell fraction in this case was 39 ± 11%, and the EPI/ICM fraction increased to 57 ± 17%. Deletion of a number of adhesion molecules is known to produce failures in PrE and EPI segregation [47,81–84]. Inhibition of the polarity determinant aPKC [53] at the mid-blastocyst stage results in a failure of PrE/EPI segregation; the increase in inappropriately localized Gata6 cells results in an increased rate of apoptosis within this population, leading to a PrE:EPI ratio of 1:1, which is within the uncertainty of our results (Fig 3).

### ΔRule 4

Deletion of the fourth rule (ΔApoptosis) resulted in 13% of embryos with a PrE precursor positioned deep within the ICM (S7 Movie). As not only misplaced PrE are likely to undergo apoptosis [42], we tested and found no significant differences in our results when we included up to 20% apoptosis in EPI cells (see S5 Fig). Despite differential adhesion and letting the system reach the equilibrium configuration, those cells were trapped in a local energy minima and could not move towards the cavity. The number of mispositioned PrE cells and, consequently, the rate of apoptosis became higher if the system did not reach equilibrium. As it is not known if the ICM cells are in equilibrium or not, our results suggest that 15% error is the lower bound estimate of how frequently differential adhesion fails to segregate PrE from EPI.

### Scaling of the blastocyst

**Fig 4:**
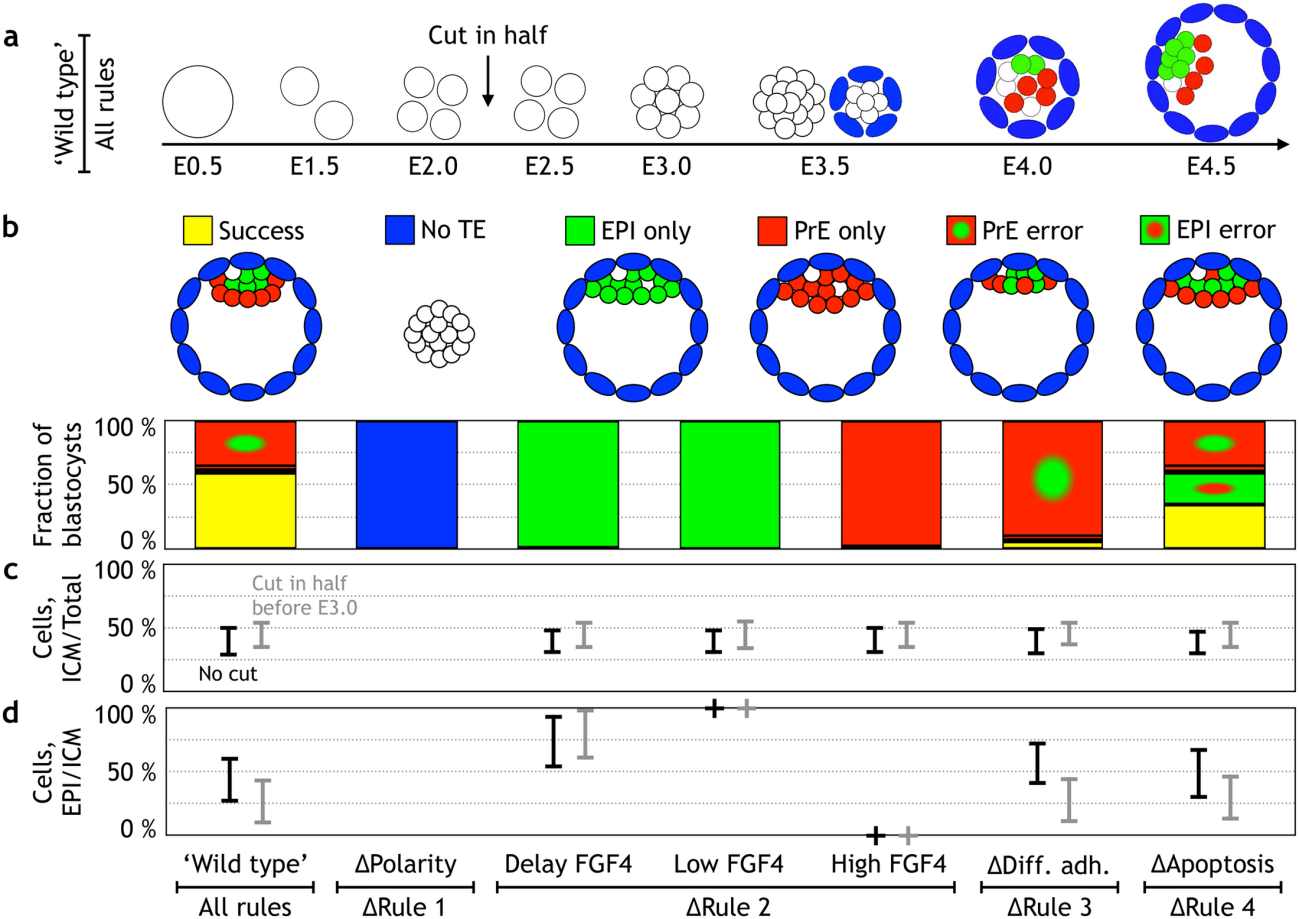
Dividing an embryo in half at 2-, 4-, or 8-cell stage results in a successful blastocyst of half the size. **(a)** Screenshots from a simulation with halving at the 8-cell stage (see S8 Movie). **(b)** Quantification of the developmental success in in silico blastocysts at embryonic day (E) 4.5 after it has been cut in half (notations and color coding are the same as in Fig 3b). **(c)** The fraction of inner cell mass (ICM) cells to the total number of cells at E4.5 in the 7 different types of embryos. **(d)** The fraction of epiblast (EPI) cells to ICM cells at E4.5. Both in (c) and (d), the number for unperturbed embryos is in black, and those that were divided in half are shown in gray. The simulation data can be found in S1 Data.

**Fig 5:**
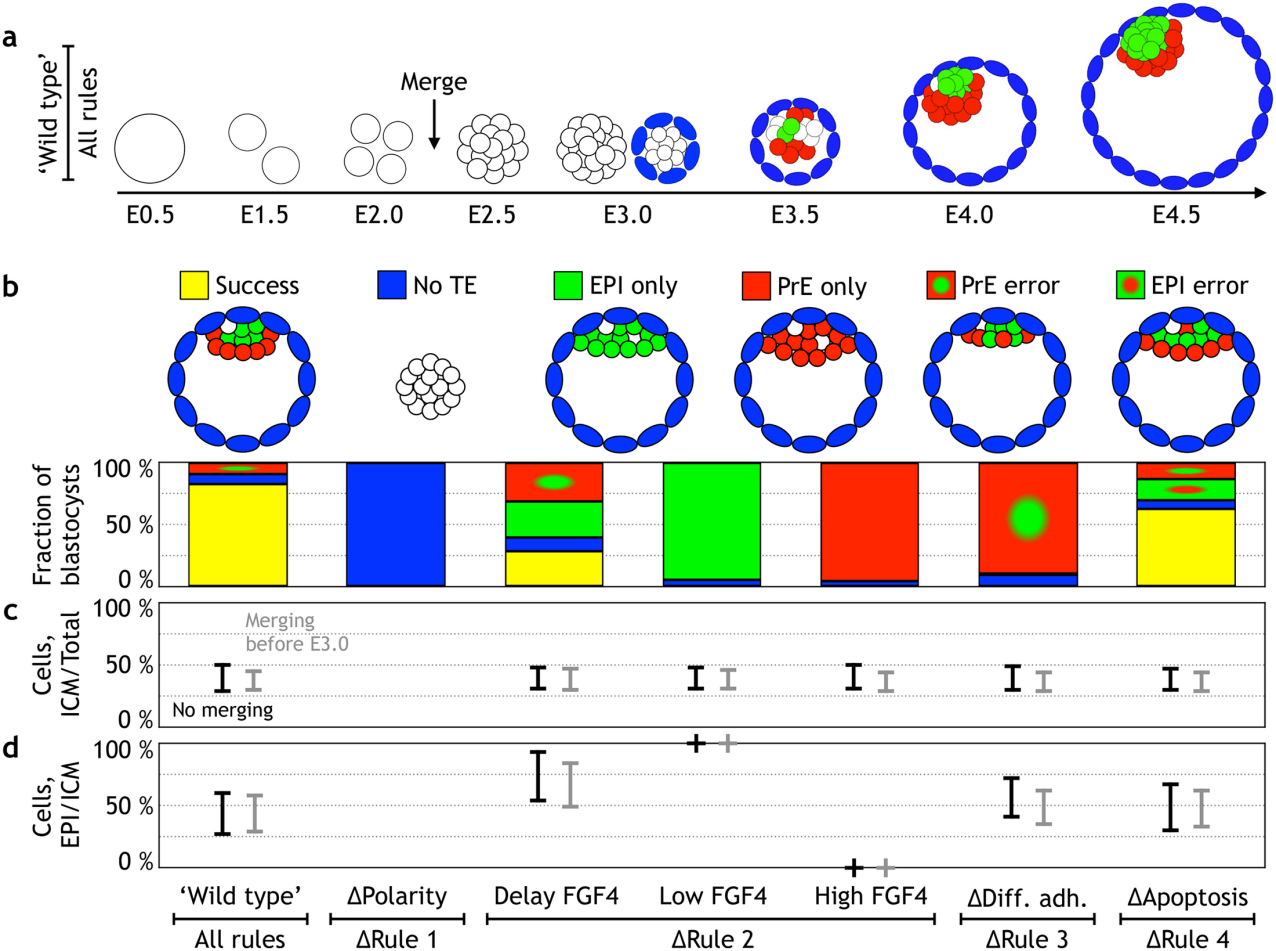
Aggregating 2 embryos at 2-, 4-, or 8-cell stage results in a successful blastocyst of double the size. **(a)** Screenshots from a simulation with merging at the 8-cell stage (see S9 Movie). **(b)** Quantification of the developmental success in in silico blastocysts at embryonic day (E) 4.5 after 2 embryos were aggregated (notations and color coding are the same as in Fig 3b). **(c)** The fraction of inner cell mass (ICM) cells to the total number of cells at E4.5 in the 7 different types of embryos. **(d)** The fraction of epiblast (EPI) cells to ICM cells at E4.5. Both in (c) and (d), the number for unperturbed embryos is in black, and the results of aggregation are shown in gray. The simulation data can be found in S1 Data.

To further validate our model, we asked if it can reproduce results of classical scaling experiments [6,67,71,85,86] in which single cells from the 2-cell stage embryo were shown to develop into blastocysts, albeit of half the size and at a lower success rate. Dividing the embryo in half at any time point up to the 8-cell stage resulted in “successful” embryos at E4.5 in about 59% of cases (Fig 4a and S8 Movie). Furthermore, halved embryos were 50% smaller (66 ± 3 cells) than the unperturbed ones (132 ± 3 cells). We also observed a 20% increase in failure rate in blastocyst formation. In our simulations, that was predominantly due to PrE error as a result of a smaller ICM and the resulting fluctuations in the ratio of PrE to EPI. In cases in which the PrE/EPI ratio is smaller than in the unperturbed embryo, there are too few PrE cells to form a layer lining EPI core, and EPI cells tend to intercalate into the PrE layer resulting in a PrE error (Fig 4b). Our model also predicts that the rate of apoptosis in successful half embryos will increase, as abolishing the apoptosis rule increases the number of failures from 15% (Fig 3b) to about 23% (Fig 4b). This is related to the increase in configurations with PrE error discussed above. The model is also consistent with the recently reported scaling results from aggregating two 8-cell stage embryos [71], see Fig 5 and S9 Movie. Thus, without any parameter adjustment, our in silico results were in complete agreement with the scaling experiments and allow us to ask which of our rules is responsible for the scaling properties of the mammalian blastocyst. As 3 out of the 4 rules (Rules 2, 3, and 4) are conditional on FGF/ERK signaling—apoptosis of PrE and differential adhesion are only possible once ICM differentiated in PrE and EPI—we asked whether stage-specific signaling competence could account for scaling.

### Scaling suggests that time from postfertilization rather than number of cells regulates FGF competence

**Fig 6:**
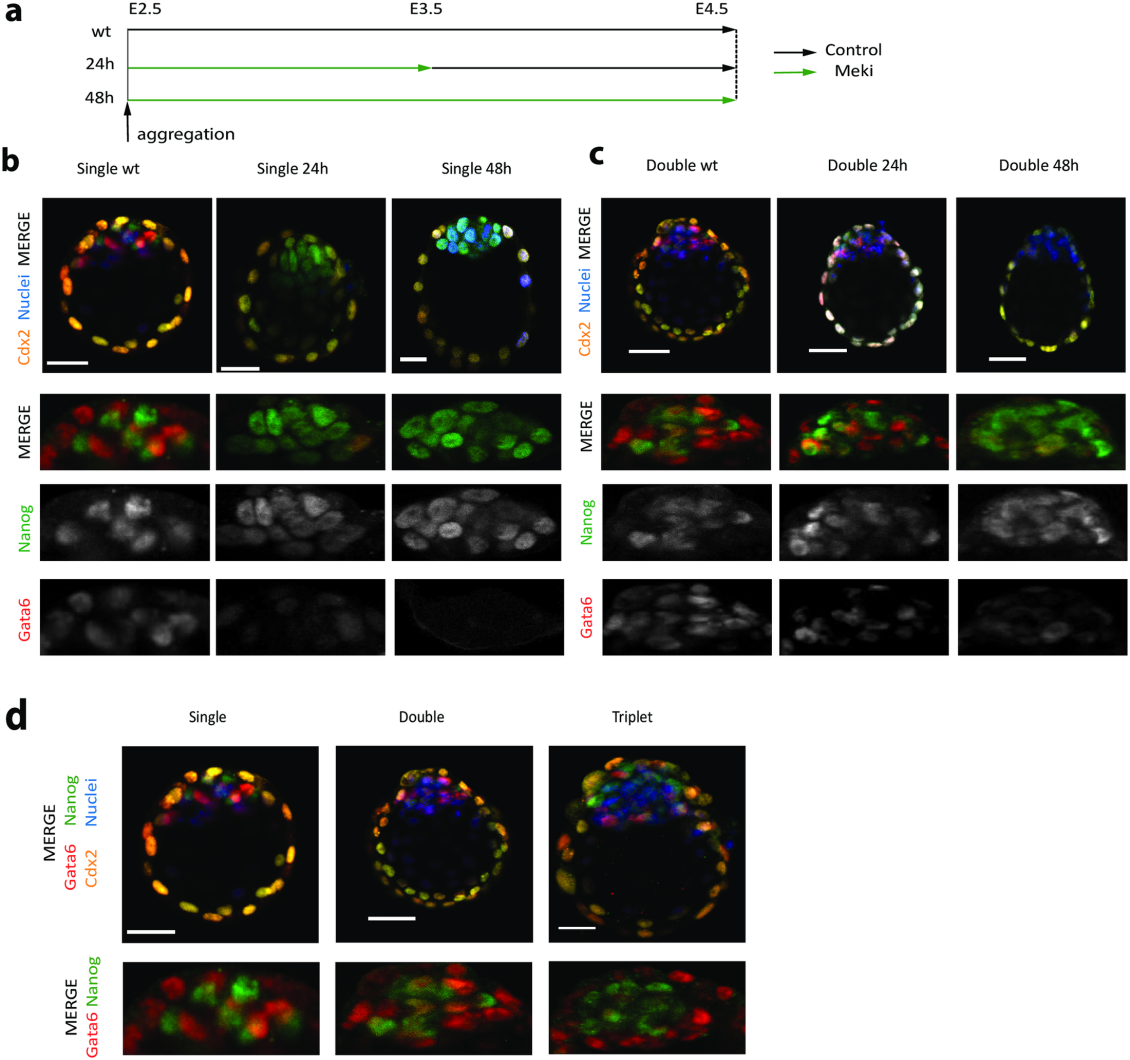
Embryo aggregation and ICM scaling in response to extracellular signal—regulated kinase (ERK) delay (inhibitor of Mek [Meki]). **(a)** Schematic illustration of the experiment. Embryos were flushed from oviducts and aggregated at the 8-cell stage (embryonic day [E]2.5), then cultured for 56 hours following manipulation. Meki was included for the first 24 hours or the entire experiment. **(b)** Immunostaining of single embryos at the completion of the experiment. Confocal optical sections through the inner cell mass (ICM) of late blastocysts immunostained for the 3 lineage markers: Nanog (epiblast [Epi]), Gata6 (primitive endoderm [PrE]), and caudal-related homeobox 2 (Cdx2) (trophectoderm [TE]). **(c)** Immunostaining of aggregated double embryos at the completion of the experiment. **(d)** Comparison of unaggregated (single), double, or triple aggregations. The length of the bar scale shown is 30 μm in all images. The data can be found in S2 Data.

In these in silico scaling experiments, we have kept the timing of ERK activation unchanged (at E3.0), which would mean that the timing of ERK activation is set at fertilization, either based on expression of the receptor or a rate-limiting factor in the pathway. When we moved ERK activation forward in time, which in effect means delaying the salt-and-pepper pattern, the blastocyst did not fully resolve the salt-and-pepper pattern by E4.5. The model also predicted that delaying ERK signaling should decrease the fraction of PrE cells (See Fig 3D “Delay FGF4” and S10 Movie). To validate this experimentally, we have performed embryo aggregation experiments with and without a potent inhibitor of Mek (PD0325901) [36,87], henceforth referred to as Meki, the kinase that responds to FGFR activation and phosphorylates ERK (Fig 6). Because of the high level of inherent stochasticity in the model and experiments, we chose to prioritize statistically significant results. The identification of a double positive (DP) fraction (through k-mean clustering, see Materials and methods) showed large fluctuations between repeat experiments; the fraction of PrE on the other hand was very robust, so we decided to focus on quantifying this cell type as an indicator ICM patterning. To set the time of FGF/ERK competence, we cultured embryos in Meki for 24 hours and then released them from the signaling block.

**Fig 7:**
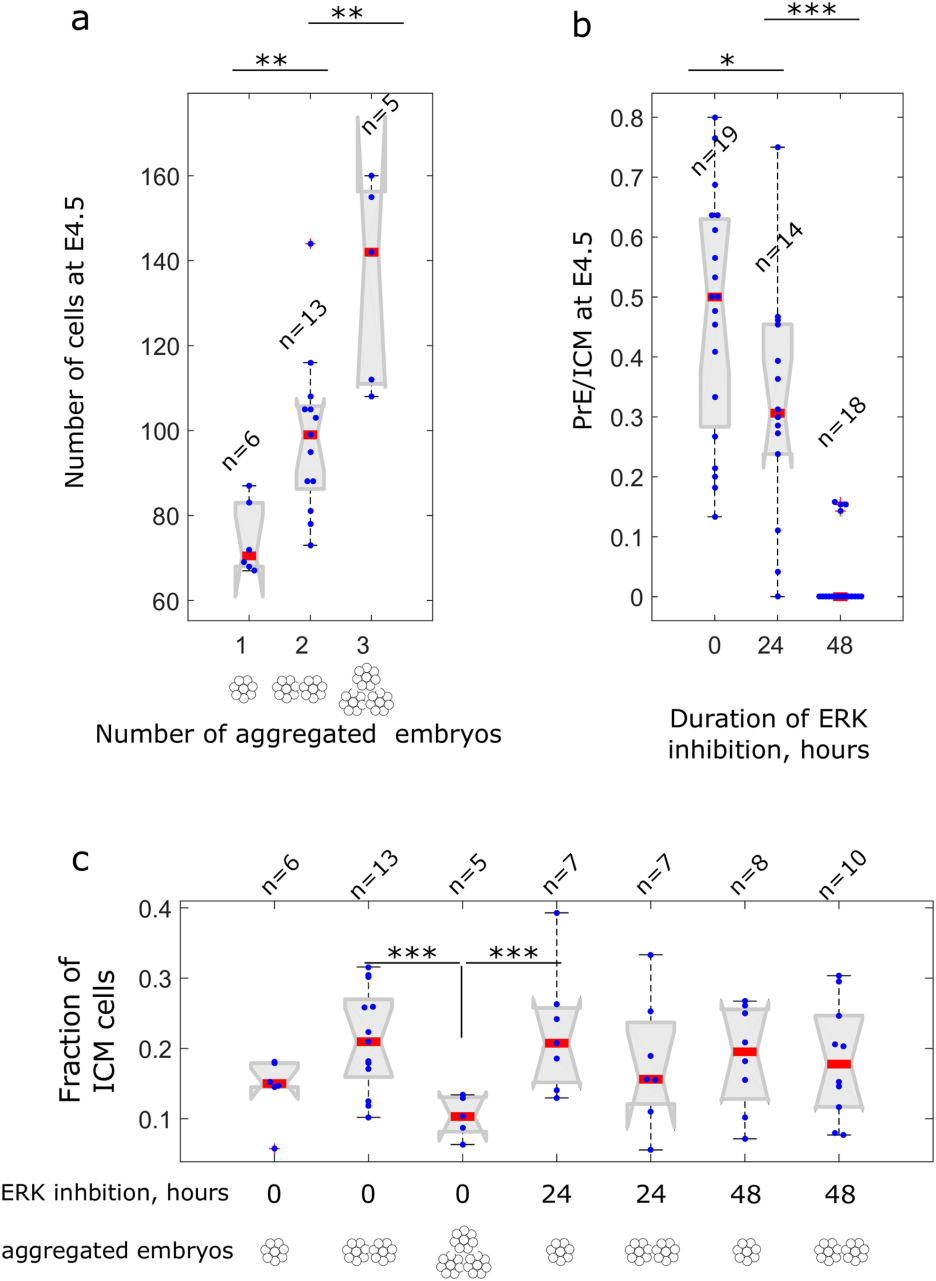
Single-cell quantification of the extracellular signal—regulated kinase (ERK) delay (inhibitor of Mek [Meki]) and embryo aggregation experiments. (a) Number of cells at E4.5 in singlets (wild type), doublets (aggregates of 2 morulas), and triplets (aggregates of three morulas). *n* denotes number of samples. **(b)** The fraction of primitive endoderm (PrE) cells among inner cell mass (ICM) at embryonic day (E)4.5 in untreated samples and samples with 24- (from E2.5 to E3.5) and with 48- (E2.5 to E4.5) hour ERK inhibition. For each case, data is pooled to include both singlets and doublets. **(c)** The fraction of ICM cells among all cells in blastocysts at E4.5. Here, the only significantly different value is for triplets; pairwise differences among the rest are nonsignificant (*p* > 0.05). Blue dots are the data points representing values for single blastocysts, and red lines mark the medians. Statistical significance was tested by nonparametric rank-sum test, see Materials and methods. Differences with *p* < 0.05 are marked by *, *p* < 0.01 are marked by **, and *p* < 0.001 are marked by ***. The data can be found in S2 Data.

In line with the model predictions, we did observe a decrease in fraction with PrE cells proportional to the duration of the ERK inhibition. Embryos were cultured for 56 hours following manipulation, to an in vitro equivalent of E4.5. During this time window, exposure to FGF/ERK signaling was manipulated in 24-hour intervals. Complete inhibition of Mek for the entire 48-hour period resulted in embryos that were entirely EPI (Figs 6b, 6c and 7b), and this is consistent with previous observations [27,71]. However, when embryos were treated for 24 hours (E2.5–E3.5) with Meki and then released from the block, PrE cells were partially recovered, but their fraction was significantly smaller than in the untreated case. Similar transient inactivation experiments have produced a variety of results [27,71] that generally support this observation but without statistical analyses of single-cell quantitation.

We also found that the duration of FGF4/ERK activation, or the point in time in which the pathway becomes competent for signaling, delimits the capacity of the ICM lineages to scale (Figs 6 and 7). Quantification of the relative level of PrE induction (Fig 6B and S4 Fig) indicates that normal aggregates maintain constant ratios of EPI/PrE and that delaying ERK activation with Meki resulted in a reduction in PrE specification. When normal and aggregated embryos are pooled, the quantitative reduction in PrE specification was statistically significant by nonparametric rank-sum test.

In addition, we observed, that based on total cell numbers, the embryos scaled but not in a perfectly linear fashion. We found that the size of the aggregated embryos increased significantly (Fig 7a), and they contained correctly proportioned ICMs, although we did observe a significantly higher ratio of TE to ICM in triplets (Fig 7c). We believe this could be a result of small differences in the relative number of founder TE cells in the triple aggregates, which proliferate at twice the rate of ICM cells [67] and could amplify these differences 2 days later. We also noticed that our embryos contain slightly lower cell numbers than the recently reported aggregation experiments [71], but we imagine these difference could be due to strain differences—in this study, we used inbred C57BL/6, whereas Saiz et al. [71] used the outbred CD1 strain.

Taken together, our attempts to delay ERK activation with Meki combined with embryo scaling experiments suggest that the accumulation of FGF4 and/or an important, limiting downstream signaling component accumulates from fertilization to the point at which this pathway can be activated. Notably, the fixed timing of cavitation, reported by Korotkevich et al. [88], suggests that the timing of TE/ICM differentiation may also start at fertilization. In our model, the timing of TE/ICM differentiation is flexible and only requires a defined time point when polarity is defined.

## Discussion

In this paper, we conceptualized our current knowledge of preimplantation development in a set of simple rules to capture the core modes of regulation, within and between cells, sufficient for successful embryonic development. The robustness of the early preimplantation development is conserved across species. The robustness and remarkable similarity in spatiotemporal patterning emerge despite stochasticity and species-to-species difference in core regulatory components [89,90]. Here, we have shown that 4 cell-based rules can explain how form can be conserved. As rules are not based on specific molecules, it explains how conservation can occur in the presence of variations in the expression and function of core members of the gene regulatory network.

We found that the in silico model, based on only 4 rules, could successfully reproduce a number of the nontrivial quantitative observations:

1. Ratios of cells in the 3 lineages: When setting the proliferation rate of ICM cells to be half that of TE, as supported by the existing data on proliferation rates [67], we obtained correct ratios of cells in all 3 lineages. With 132 ± 3 cells at E4.5, the ratio of ICM/total cell number is 39 ± 12%, and the EPI/ICM fraction is 44 ± 18%, which both agree with the experimental values reported by Artus et al. [48] and Saiz et al. [53] (Fig 3c and 3d).
2. Frequency of apoptosis inside the ICM: Modeling results predict that differential adhesion fails to segregate PrE from EPI cells in 15% of embryos. In these embryos, PrE cells remain deep inside the ICM and undergo apoptosis. These estimates are in a good agreement with the numbers reported by Plusa et al. [4], in which in 22% (27 out of 123) of the embryos, PrE cell were observed inside the ICM, and about half of them underwent apoptosis.
3. Outcomes of scaling experiments: Halving embryos at 2-cell stage was reported to result in embryos of about half the size and a decreased success rate [6,85]. The in silico realization of this experiment matches both the reported size scaling of the embryo and, importantly, the decreased success rate (by 20%) exceptionally well.

In addition to these quantitative observations, our model also suggests that specific criteria within the model may be responsible for different aspects of blastocyst development.

### Polar interactions of TE cells are necessary for cavity formation

Without polar interactions we could not form the cavity. These results are in a good agreement with the experiments reporting on the consequences of strong polarity defects [72–76]. In earlier in silico models, which did not include polarity, the cavity was introduced by hand and was assumed to grow and create a positive pressure on TE thus driving their cell division [59,58]. In contrast, in our model, the growing cavity is a consequence of dividing TE cells, which form a shell-like layer due to polar interactions. While our results do not rule out the osmotic expansion of the blastocyst, they argue that the expansion by TE proliferation should be considered on equal footing. It is hard to delineate which of the 2 is a driving mechanism as the 2 are tightly coupled. First, even if the blastocyst expands by TE proliferation, the cavity’s osmotic pressure should be maintained at homeostasis. Second, drugs inhibiting TE ion channels do not solely act to decrease blastocyst expansion but also perturb TE metabolism [91] and proliferation. To maintain homeostasis during blastocyst expansion, it is likely that the 2 mechanisms act in tandem, feeding back on each other.

### Model predicts that a Turing-like mechanism in FGF/ERK signaling assures the right PrE/EPI cell proportions

When seen from the perspective of one of the markers, e.g., Nanog in EPI cells, the reduced scheme of FGF/ERK signaling (Fig 2 and S2 Fig) can be generalized to a Turing-like patterning mechanism. This mechanism is known for patterning of animal fur, e.g., emergence of black spots in leopards, and is often summarized as “local amplification and global inhibition.” In the case of ICM cells, the “local amplification” results from Nanog intracellular positive feedbacks, whereas the “global inhibition” is realized by Nanog cells secreting FGF4 and inhibiting Nanog in neighboring cells (S2 Fig). Thus, as long as FGF4 is produced in predetermined ICM cells, the pattern of intermixed cell types will automatically emerge. However, when we sampled early stages of blastocyst formation, we found that the patterning of the early ICM (at E3.5) was similar, but not identical, to the recently reported experiments on quantification found in Saiz et al., 2016. In particular, we never detected EPI cells induced in the absence of PrE cells (S6A Fig).

Although recent single-cell RNA sequencing data [39,92] suggests that undetermined ICM cells do express FGF4, they appear to do so to a lesser extent than EPI cells, and this was not accounted for in our original model. We therefore decided to test how this may influence our modeling results; we modified the model to make undetermined ICM cells contribute half as much FGF4 as determined EPI cells. We found that the fractions of cells at E4.5 did not change (S6 Fig), and that we generated normal blastocysts, indistinguishable from our previous simulations. However, this slight modification now recovers the subpopulation of embryos with EPI but no PrE at E3.5 reported by [71] (S6A Fig). Thus, our model not only generated correctly patterned blastocysts, but now also reproduces the earliest phases of lineage segregation with higher fidelity. These simulations make a new, experimentally verifiable prediction that unsegregated ICM cells express lower, but functional, levels of FGF4 than differentiated EPI. It also further demonstrates the importance of self-regulating dynamics in patterning the blastocyst, demonstrating that the final result is not sensitive to initial conditions. The capacity to vary initial conditions without impacting on the final result also provides insight into parameters that can be manipulated in evolution.

The FGF/ERK pathway coupled to Nanog/Gata positive feedback was proposed to control PrE/EPI cell proportions in 2 other models, one exploring isolated ICM patterning [57] and the other PrE/EPI specification in embryonic stem cells (ESCs) [93]. Neither model captures the dynamic geometry of the growing embryo. Our model incorporates a conceptualized FGF/Nanog/Gata feedback circuit into embryonic development, showing that a form of this mechanism can function in the highly dynamic environment in which cells divide and move due to differential adhesion.

As a consequence of this “local amplification, global inhibition,” PrE and EPI cells in the model are capable of changing their identity at all times during the FGF/ERK competence window, i.e., the pathway remains active. While in ES cell culture and in FGF4 manipulation experiments by Bessonnard et al. [57] and Yamanaka et al. [27] there is a window in which cells are observed to change their identity, this does not seem to occur frequently under physiological levels of FGF4 in unperturbed blastocysts [70]. It is, however, not known if these changes do not occur because cells are not capable of switching or because they reach an appropriate configuration in which the switching is not required.

Our simulation suggest that the latter explanation is correct, and this explains why the cells of the blastocysts remain competent to undergo regulative transformations in response to signaling manipulation while maintaining an apparently deterministic trajectory in normal development. This, and to what extent the number of FGF4-secreting neighbors determines the fate of the cell, can be tested through targeted laser ablation of cells such as to shift the balance between FGF4-secreting and nonsecreting cells in the neighborhood.

### Apoptosis proofreads the outcome of differential adhesion

Simulation results suggest that differential adhesion alone can often (62% of embryos) be sufficient for correct spatial arrangement of PrE and EPI cells. It is believed that the position-dependent apoptosis of Gata6 cells may play an important role in resolving the occasional positional errors: Plusa et al. [4] reports that the isolated Gata6 cells deep inside the ICM apoptose 6-fold more often than the correctly positioned PrE cells facing the blastocoel. One possible mechanism for the positional difference in apoptosis is if the concentrations of the cytokines LIF [50,51] and PDGF [52,53]—known to promote PrE survival—are lower inside the ICM than at the junction of the EPI, PrE, and blastocoel. While this still remains to be tested experimentally, the current knowledge on LIF is in line with this hypothesis. LIF secreted from TE is likely to accumulate to higher concentrations at the PrE/blastocoel boundary as there are more TE cells facing the blastocoel, and LIF produced by these cells can diffuse freely until it reaches the PrE layer.

Our observations indicate apoptosis is important for the robustness of pattern formation. The model predicts that apoptosis becomes increasingly important as the difference between adhesive properties is reduced. The difference in adhesion properties could vary in different genetic backgrounds and also must vary in time as differentiation progresses. In cases in which there is flexibility in adhesion, there would be a greater requirement for apoptosis in proofreading.

While we have shown that differential adhesion in combination with apoptosis are sufficient for proper lineage segregation, a number of other mechanisms may contribute to robustness in the segregation process. First, it has been suggested that cellular movements involve not only passive but also active mechanisms, associated with cell protrusions [42]. In line with this, in silico studies that did not consider apoptosis, showed that failures in segregation can be reduced if differential adhesion is complemented by directed cell movements [59]. Second, PrE differentiation occurs in stages that include an uncommitted and biased state within the ICM and committed, PrE lining the blastocoel. Thus, when tested in heterotrophic grafting experiments, early PrE progenitors within the ICM were competent to make EPI, while PrE progenitors that line the blastocoel cavity are only able to participate in endoderm development [29].

In our simulations—in which the segregation of the PrE and EPI is based on differential adhesion—we often, in about 50% of simulations for wild type, observe PrE forming 2 layers. While this is obviously in contrast with the observed single PrE layer in real embryos, to keep the model simple, we choose to count them as a success as long as the PrE layers seal EPI core from blastocoel. Expanding the model to include polar interactions between PrE cells lining the cavity would ensure a single PrE layer and provide a contiguous barrier between the EPI and the cavity [53].

### Model predicts the invariant timing of the FGF/ERK signaling is necessary for blastocyst scaling

Scaling presents a fascinating example of the robustness in embryo development, and the experimental manipulation of this phenomena served as an important validation step for the model. The close match between the experimental observations and our simulation predicted that timing of FGF/ERK signaling may be the key parameter for controlling the scaling outcome. With the 4 rules in place, the embryo would scale when divided in half or doubled prior to compaction. However, we only observe this property if cell fate specification and emergence of the salt-and-pepper pattern—attributed to the FGF/ERK signaling—take place at the same time counted from fertilization. This implies that competence for FGF/ERK signaling is primed for activation from fertilization. We have validated this prediction by showing that the ratio of PrE in the ICM decreases upon transient inhibition of FGF4 signaling both in the model and in cultured embryos.

The notion that we observe normal development with 4 rules that are largely independent of the initial gene regulatory network is particularly relevant to the current debate about the extent to which stochastic gene expression governs the initiation of blastocyst development. Our model demonstrates that initial differences in stochastic gene expression are not a necessary prerequisite for the generation of 3 distinct lineages. Instead, differentiation emerges based on the responses of a cell to its local environment, as interpreted via differential proliferation, adhesion, and gene expression. The existence of a set of rules that allow for blastocyst formation as long as a few simple conditions are satisfied could be an enabler of stochastic variation. It also could explain how mammalian development can allow for the fundamental changes in the gene regulatory network that have been observed when single-cell sequencing data has been compared between mouse and human [89,94,95].

## Materials and methods

For simplicity, we implemented our model in 2D. Cells are modeled as circles with the radius set to one.

### Model

To compare with experimental results from 3D blastocysts, we used simple scaling relationships converting between 2D and 3D. Thus, the number of TE cells, $N_{TE}^{3D}$, placed on the surface of the sphere would correspond to $N_{TE}^{2D}=\sqrt{\pi N_{TE}^{3D}}$ in 2D. Similarly, for cells in the bulk: $N_{EPI/PrE}^{2D}={\left(\frac{3}{4}N_{EPI/PrE}^{3D}\sqrt{\pi }\right)}^{2/3}$.

### Finding nearest neighbors in 3D

- For each cell *i*, list its 20 closest neighbors (20 *j*'s) based on center-to-center distance.
- Find the point halfway between *i* and each *j*.
- If the halfway point is closer to another cell (among the 20) than *i*, then it is not cell *i*'s nearest neighbor.

### Interactions between cells

The interaction between 2 cells is given by the following potential, *V* (see Fig 2c):

V(d)=exp(−d)−S exp(−d/β) (2)

where *d* is the distance between the cells, *S* is the attraction factor given by Eq 1, and *β* is the parameter controlling the range of the attraction.

This potential assures repulsion at short distances, i.e., 2 cells separated by a distance less than 1 cell diameter (*d* < 2) will repulse from each other. On the other hand, if cells are separated by more than 1 cell diameter (*d* > 2), they will be attracted to each other. In the simulations, we set a distance cutoff, setting potential to 0 for all cell pairs that are further away than 5 cell radii. *S* and *β* are chosen to produce a tight packing of cells with minimal overlap (see configuration of cells in Fig 2a, with *S* = 0.6 and *β* = 5). The choice of these parameters as well as the form of the potential are not important for the model outcome as long as the condition above is satisfied. While we keep *β* = 5 fixed throughout the simulations, the *S* will capture lineage-specific differences in adhesive properties and is thus a lineage-specific parameter. Prior to first cell-lineage decision at E3.0, cells are assumed to have the same adhesive properties and thus the same strength of attraction, *S* = 0.6.

The motion of the cells is described by the overdamped equation of motion:

dx/dt=−dV/dx+η (3)

where *dV*/*dx* is a *x*-projection of the resulting force from all of the pairwise cell—cell interactions.

To ensure that system reaches equilibrium, we add the noise term *η*. In the simulations, this is implemented by adding a random number from a normal distribution with a mean of 0 and a standard deviation of 10^−3^ at every time step. The equation is integrated numerically using Euler integration scheme. The *y*-position is determined in a similar way.

The polarity of the TE cells is assumed to be affected by the orientation of the polarities in neighboring TE cells such that they tend to point in the same direction. This is well described by a pairwise polarity potential *V*_*p*_ = −cos *θ*_*i*,*j*_, where *θ*_*i*,*j*_ = *α*_*i*_ − *α*_*j*_ measures the angle between the polarities of the *i*,*j* TE neighbors.

The orientation of the polarity is described by an angle *α* and, similarly to the equation above, the change in polar orientation is given by $\frac{d\alpha }{dt}=-0.1\frac{dV_{p}}{d\alpha }+\eta_{p}$ where the prefactor of 0.1 makes the changes in polarities happen slower compared to the changes in the positions. This is necessary for the stability of the system. The noise term is implemented in the same way as above, with the only difference being that the random numbers are multiplied by π.

The TE is noise sensitive. With the chosen standard deviation 10^−3^ on the noise parameter, the simulation is very sensitive to the factor 1.4 in Eq 1. If this factor is increased to 1.5, the TE repulses too much when a new TE cell is added. On the other hand, if the factor is decreased to 1.3, it becomes too weak and cannot keep the TE together at E4.5. Thus, here, we apply the maximum tolerated noise to the system. Less or even no noise is also acceptable, and it allows the factor in Eq 1 to be decreased.

### Growth of the blastocyst

We started with 1 cell. In contrast to an earlier model where the growth of the blastocysts was driven by the growing blastocoel, in our simulations, the blastocyst grows as a result of cell division: A cell is randomly selected to undergo division, and the daughter cell is positioned between the mother cell and the nearest neighbor cell. In real blastocysts, prior to division, cells gradually increase in size, allowing other cells to readjust their position such that the system is near equilibrium at all times. For simplicity, we chose to keep cell size constant; however, that results in strong perturbation of the equilibrium during the simulated cell division. To assure that the configurations of the simulated blastocysts are not affected by this, we allowed enough time for the system to relax before the next cell division. In 3D, one can significantly speed up the simulations by introducing a new cell in the center of the 3 nearest neighbors, as this is closer to the minimal energetic configuration and reduces the number of relaxation steps.

### Growth and specification during E3.0 to E4.0

During implementation of Rule 2, we first pick up random cells to divide among the undetermined ICM’s. At the division, the likelihood of a cell to convert to PrE is proportional to the fraction of high FGF4 (EPI or undetermined ICM) cells in the neighborhood ($P\left(PrE\right)=\frac{\#\;of\;high\;FGF4\;neighbors}{\#\;of\;ICM\;neighbors}$); conversely, the likelihood to convert to EPI is *P*(*EPI*) = 1 − *P*(*PrE*). After all cells have specified, a random ICM is chosen to divide, and at the division, the same rule applies as for undetermined ICM’s.

### Mouse maintenance

Embryos used in this study are inbred C57Bl/6NRj (Janvier Labs, France).

Mice were maintained in a 12-hour light/dark cycle in the designated facilities at the University of Copenhagen, Denmark. Embryo donor females underwent super-ovulation treatment following a standard protocol: intraperitoneal injection (IP) of 5 IU PMSG (Sigma) per female and IP injection of 5 IU hCG (Chorulon, Intervet) 47 hours later, followed by overnight mating with C57Bl/6NRj stud males. The following morning, females were monitored for copulation plug formation. Embryos were considered E0.5 on the day of plug detection. Animal work was carried in accordance with European legislation and was authorized by and carried out under Project License 2012-15-2934-00743 issued by the Danish Regulatory Authority.

### Embryo collection and generation of double and triple embryos

Embryos were obtained at 8-cell morula stage by washing E2.5 oviducts with M2 medium (Sigma). In order to remove the zona pellucida, morulae were briefly incubated in Acid Tyrode’s solution (Sigma) at RT and then washed in M2 medium.

To generate aggregates, embryos were placed in pairs or triplets in aggregation microwells made with an aggregation needle (BLS) on Petri dishes in KSOM medium (LifeGlobal) drops. Drops were overlaid with mineral oil (Nidoil, Nidacon). Single embryos were placed alone as control. KSOM was supplemented with 0.1% BSA (Sigma) to avoid embryos adhering to the plastic. Embryos were cultured at 37°C, 5% CO^2^ and 90% relative humidity. For MEK inhibition treatment, 1 μM of PD 0325901 (PZ0162, Sigma) was diluted into KSOM. Wild-type embryos were generated by culture in KSOM. 24-hour and 48-hour treated embryos were generated by culture in KSOM with PD 0325901 for 24 hours and 48 hours, respectively. The data were collected over 4 repeat experiments.

### Embryo staining and imaging

Fifty-six hours after aggregation, embryos at E4.5 were fixed in 4% PFA solution for 15 minutes at room temperature. Afterwards the embryos were stained as previously described [96]. The primary antibodies used were: anti-Nanog (eBioscience, 14–5761; 1:200), anti-Cdx2 (Biogenex, MU392A-UC; 1:200), and anti-Gata6 (R&D, AF1700; 1:100). Embryos were imaged in an Attofluor chamber (ThermoFisher) on a 25-mm glass coverslip using 10x magnification on a Leica TCS SP8 confocal microscope.

### Single-cell quantification

We used ImageJ to manually track positions of the nuclei in single cells. Positions were saved and intensities for each fluorescence channel at each position were processed by custom-built Matlab scripts (available upon request). For each of the channels, we have used the mean intensity of the 5 x 5 x 3 voxel as a readout for single cell. To filter out the noise, cells with the Dapi intensity below 1 were removed. To differentiate between TE and ICM cells, for each of the cells, we ranked the intensities of Cdx2, Nanog, and Gata6. Cells where Cdx2 ranked first, were classified as TE cells. We validated that the identified TE cells localize to the periphery of the embryo. We classify the ICM cells as described in Saiz et al. [71]: First, we performed k-means clustering (by Squared Euclidean distance metric, Matlab built in function) on the log(Gata6) and log(Nanog) into 3 clusters with 10 repetitions on all data pooled together. Second, we classified high Gata6 and high Nanog cells as DP cells, high Gata6 and low Nanog as PrE cells, and low Gata6 and high Nanog as EPI cells. See S3 Fig for the results of the clustering.

### Statistical tests

As the distributions of the analyzed properties were clearly far from normal, we used nonparametric rank-sum test to estimate the statistical significance of the difference in medians.

## Supporting information

S1 Fig ICM in the absence of TE. The model predicts that PrE (red) will surround the EPI core (green) due to differential adhesion. This is in agreement with experimental data by Canham et al. (2010) (see also S3 Movie).
(TIF)

S2 Fig Turing mechanism behind scaling of PrE (red) and EPI (green) cells. a. Schematic representation of local, intracellular positive feedback and global, intercellular inhibition. Nanog (or Gata6) positive feedback is a result of mutual inhibition between Nanog and Gata6. Nanog-high cells secrete FGF4 and thereby inhibit Nanog in neighboring cells resulting in “global”, intercellular inhibition. b. Snapshot of the simulation of an “infinitely” large ICM with 10x10 cells with periodic boundary condition interacting with the Rule 2 starting from undetermined ICM. c. Corresponding time-course showing that the ratio of PrE/ICM is stable and converges to 0.5 independent of ICM size.
(TIF)

S3 Fig Results of k-means clustering. Generated using the S2 Data.
(TIF)

S4 Fig Fraction of PrE to all ICM cells under different aggregation and delay conditions. Singles are marked by 1X and doublets by 2X. WT stands for no treatment, 24h and 48h mark duration of treatment with MEKi inhibitor starting at E2.5. n denotes number of embryos. Figure is generated using the S2 Data.
(TIF)

S5 Fig Testing for impact of apoptosis in any of the ICM cells. Results of simulations where in addition to our Rule 4, 20% of EPI cells undergo apoptosis at E4.5. This modification results in no significance difference with the earlier results when only misplaced PrE cells undergo apoptosis (compare with Fig 3). The data used to generate the figure is in S2 Data.
(TIF)

S6 Fig Assessing the impact of differences in FGF4 production in undetermined ICM and defined EPI cells. **A**. Results of the model described in the main text, where undetermined ICM and EPI are assumed to contribute equal amounts of FGF4. **B**. Results of simulations where undetermined ICM’s contribute half as much FGF4 as EPI. In these simulations, we observed alterations in the initial specification of Epi and PrE at E3.75. Embryos with EPI, but no PrE (in addition to undetermined ICM) appear only in **B**, but the results at E4.5 are the same in both cases, with ratios converging to 50%. **C**. and **D**. The means (boundaries of shaded regions) and medians (notches of the box-plots) of the fraction of cell types are shown for simulations shown in **A**. and **B**. The data used to generate this figure is in S3 Data.
(TIF)

S1 Data Results of the simulations, used to generate Figs 3–5. Data used to generated Figs 3–5 and S5 Fig.
(XLSX)

S2 Data Single cell data used to generate Figs 6 and 7. Data used to generate Figs 6 and 7 and S3 and S4 Figs.
(XLSX)

S3 Data Data used to generate S6 Fig. (XLSX)

S1 Movie Example of a successful development of an *in silico* blastocyst when all four rules applied (see also Figs 1 and 3a). (MOV)

S2 Movie 3D example of the successful development of an *in silico* blastocyst when all four rules are applied. The development is initialized at the 3-cell stage and goes up to 116 cells at E4.5. As in 2D, two TE cells in the simulation correspond to one real TE cell. In addition, TE cells are here shown with ¼ the size of ICM cells in order to be able to view the ICM. At E4.5, the PrE completely covers the EPI.
(MOV)

S3 Movie *In silico* development of the ICM in the absence of the TE. Notice the emergence of the salt-and-pepper pattern and the effect of differential adhesion which finally results in S1 Fig.
(MOV)

S4 Movie Example of a development of an *in silico* blastocyst depleted of FGF4. The ICM consists of only Nanog expressing cells (see also Fig 3b–3d).
(MOV)

S5 Movie Example of a development of an *in silico* blastocyst in excess of FGF4. The ICM consists of only Gata6 expressing cells at the late blastocyst stage (E4.5). (see also Fig 3b–3d).
(MOV)

S6 Movie Example of a development of an *in silico* blastocyst in the absence of differential adhesion (ΔDifferential adhesion case). Many Gata6 cells are misplaced at E4.5 and therefore undergo apoptosis. As a result, the fraction of ICM/total cell number is smaller than in the WT.
(MOV)

S7 Movie Example of a development of an *in silico* blastocyst in the absence of apoptosis (ΔApoptosis case). Gata6 expressing cells are misplaced in about 13% of the embryos, ending between the TE and the EPI at E4.5 (this is referred to as an EPI error).
(MOV)

S8 Movie Cutting the two-cell morula in half, results in a “successful” blastocyst of half-size compared to the wild type at E4.5. In the movie, cutting in half is performed at the 8-cell stage (E2.5) resulting in the half number of cells at that stage (see also Fig 4a).
(MOV)

S9 Movie Merging two morulae before polarity is developed, results in a double blastocyst size compared to the wild type at E4.5. In the movie, merging of the two morulae is performed at the 8-cell stage (E2.5) resulting in the double number of cells at that stage (see also Fig 5a).
(MOV)

S10 Movie Example of a development of an *in silico* blastocyst with delayed FGF4 expression. Since FGF4 is needed for Gata6 expression, this mutant results in a decrease in the number of PrE cells (see also Fig 3b–3d).
(MOV)

## Abbreviations

aPKC: atypical protein kinase C
Cdx2: caudal-related homeobox 2
DP: double positive
dsRNA: double-stranded RNA
E: embryonic day
EPI: epiblast
ERK: extracellular signal–regulated kinase
ESC: embryonic stem cell
FGF: fibroblast growth factor
FGFR2: FGF receptor
ICM: inner cell mass
LIF: Leukemia inhibitory factor
Meki: inhibitor of Mek
Oct4: octamer-binding transcription factor 4
PDGF: Platelet-derived growth factor
PrE: primitive endoderm
RNAi: RNA interference
siRNA: small interfering RNA
Sox2: sex-determining region Y-box 2
TE: trophectoderm
Tead4: TEA domain family member 4
YAP: Yes-associated protein 1
