# Supplementary figures and images for: Four simple rules that are sufficient to generate the mammalian blastocyst
Source: PLoS Biol. 2017 Jul 12;15(7):e2000737. doi: 10.1371/journal.pbio.2000737 (PMC5507476; doi:10.1371/journal.pbio.2000737)

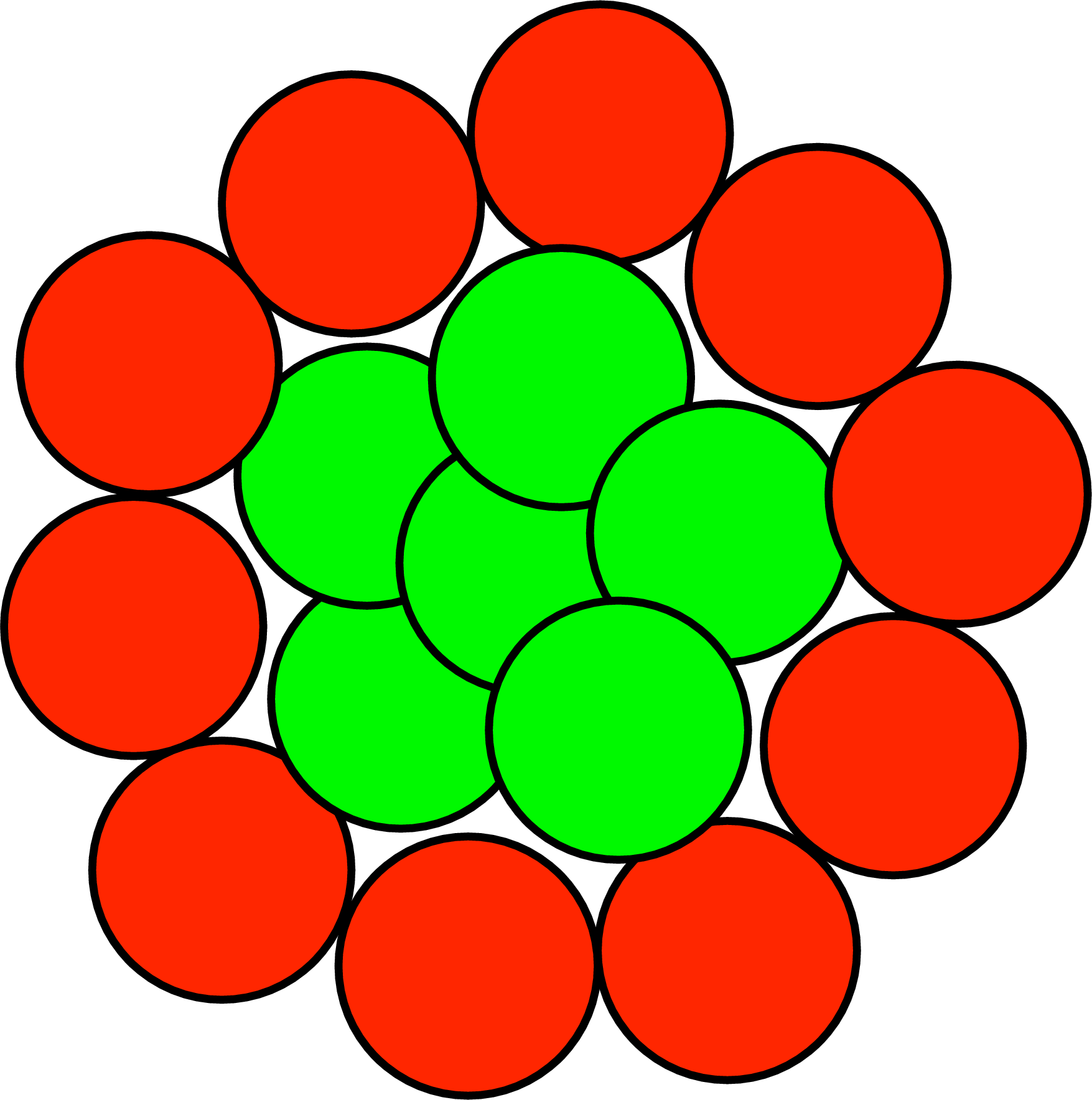

Supplement: S1 Fig — The model predicts that PrE (red) will surround the EPI core (green) due to differential adhesion. This is in agreement with experimental data by Canham et al. (2010) (see also S3 Movie). (TIF) [file pbio.2000737.s001.tif]

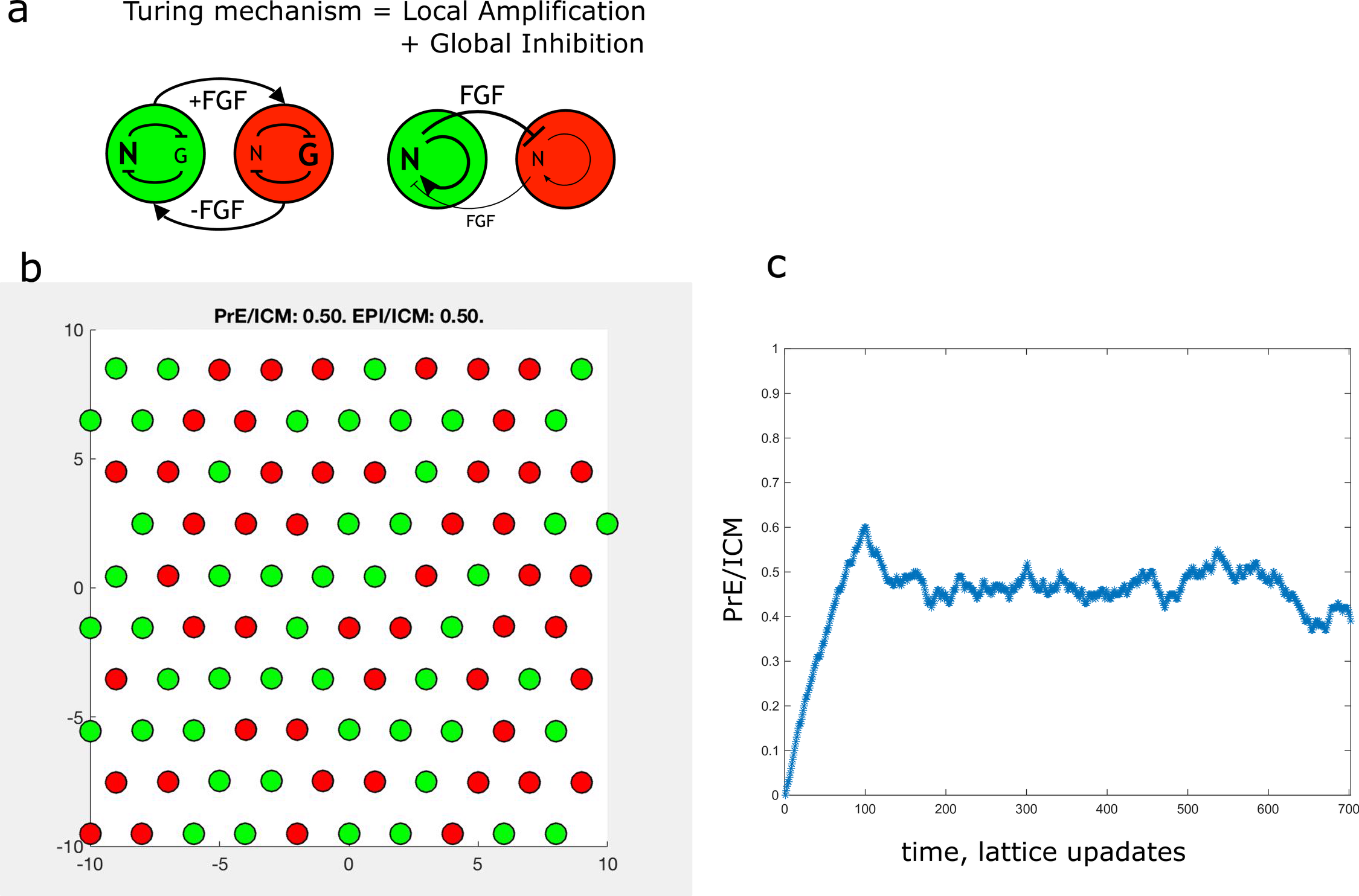

Supplement: S2 Fig — a. Schematic representation of local, intracellular positive feedback and global, intercellular inhibition. Nanog (or Gata6) positive feedback is a result of mutual inhibition between Nanog and Gata6. Nanog-high cells secrete FGF4 and thereby inhibit Nanog in neighboring cells resulting in “global”, intercellular inhibition. b. Snapshot of the simulation of an “infinitely” large ICM with 10x10 cells with periodic boundary condition interacting with the Rule 2 starting from undetermined ICM. c. Corresponding time-course showing that the ratio of PrE/ICM is stable and converges to 0.5 independent of ICM size. (TIF) [file pbio.2000737.s002.tif]

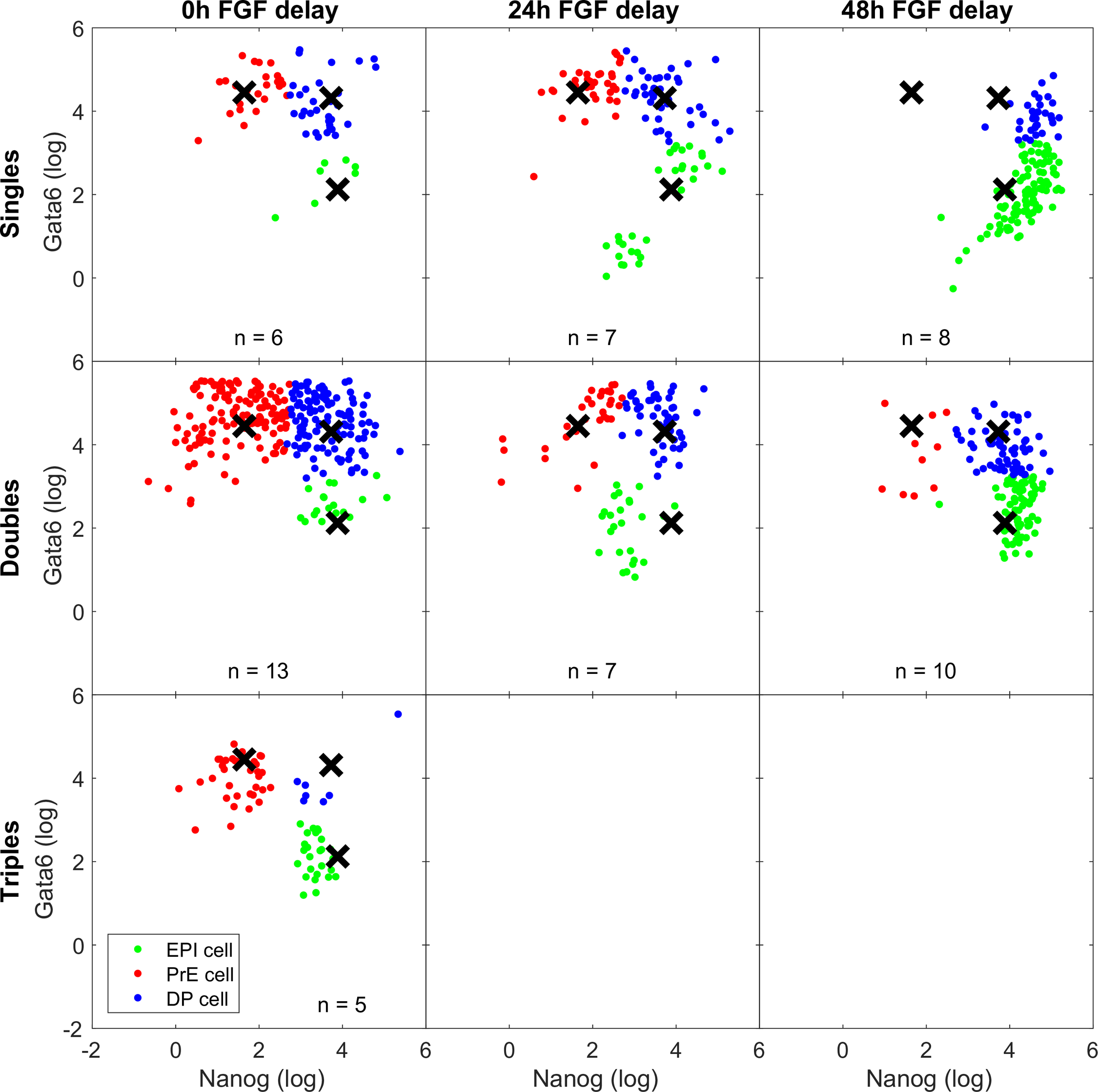

Supplement: S3 Fig — Generated using the S2 Data. (TIF) [file pbio.2000737.s003.tif]

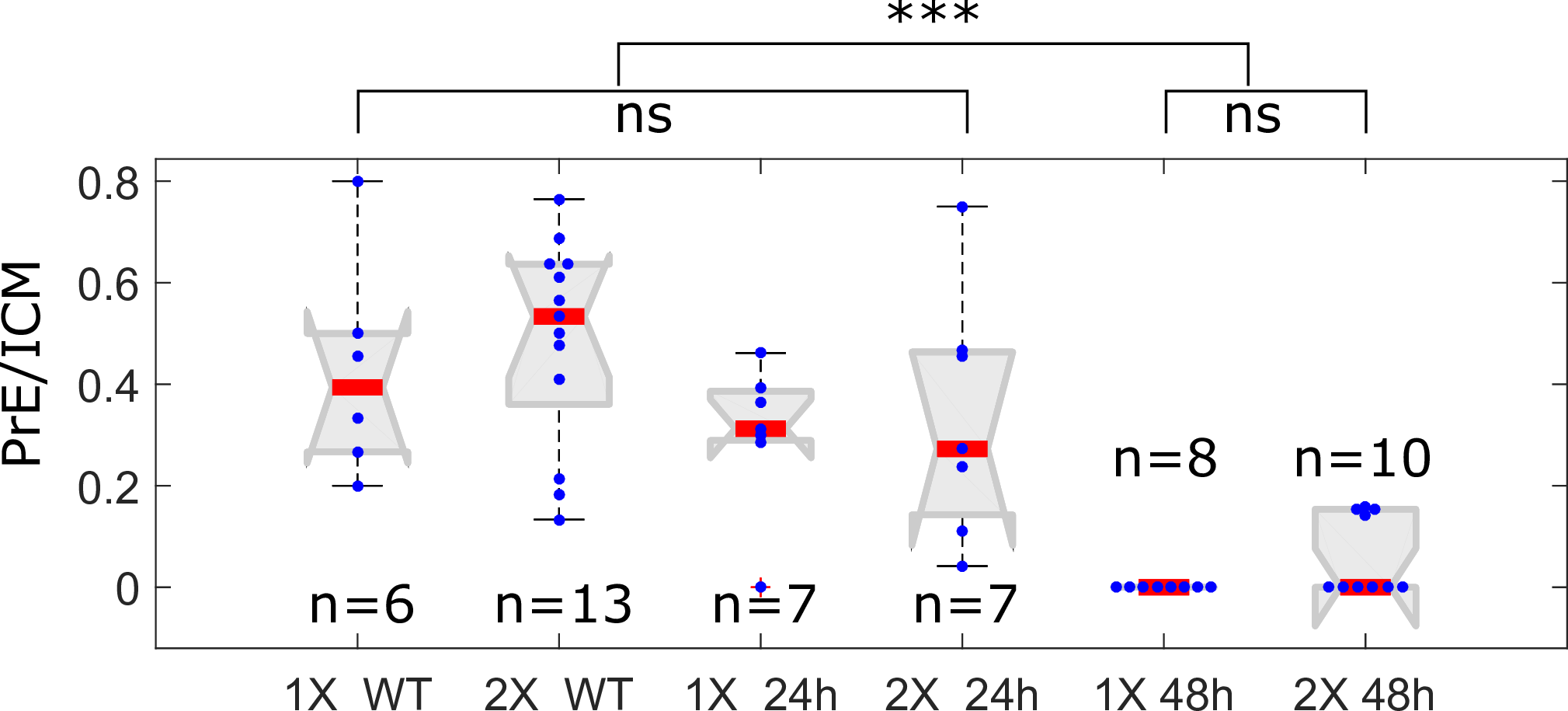

Supplement: S4 Fig — Singles are marked by 1X and doublets by 2X. WT stands for no treatment, 24h and 48h mark duration of treatment with MEKi inhibitor starting at E2.5. n denotes number of embryos. Figure is generated using the S2 Data. (TIF) [file pbio.2000737.s004.tif]

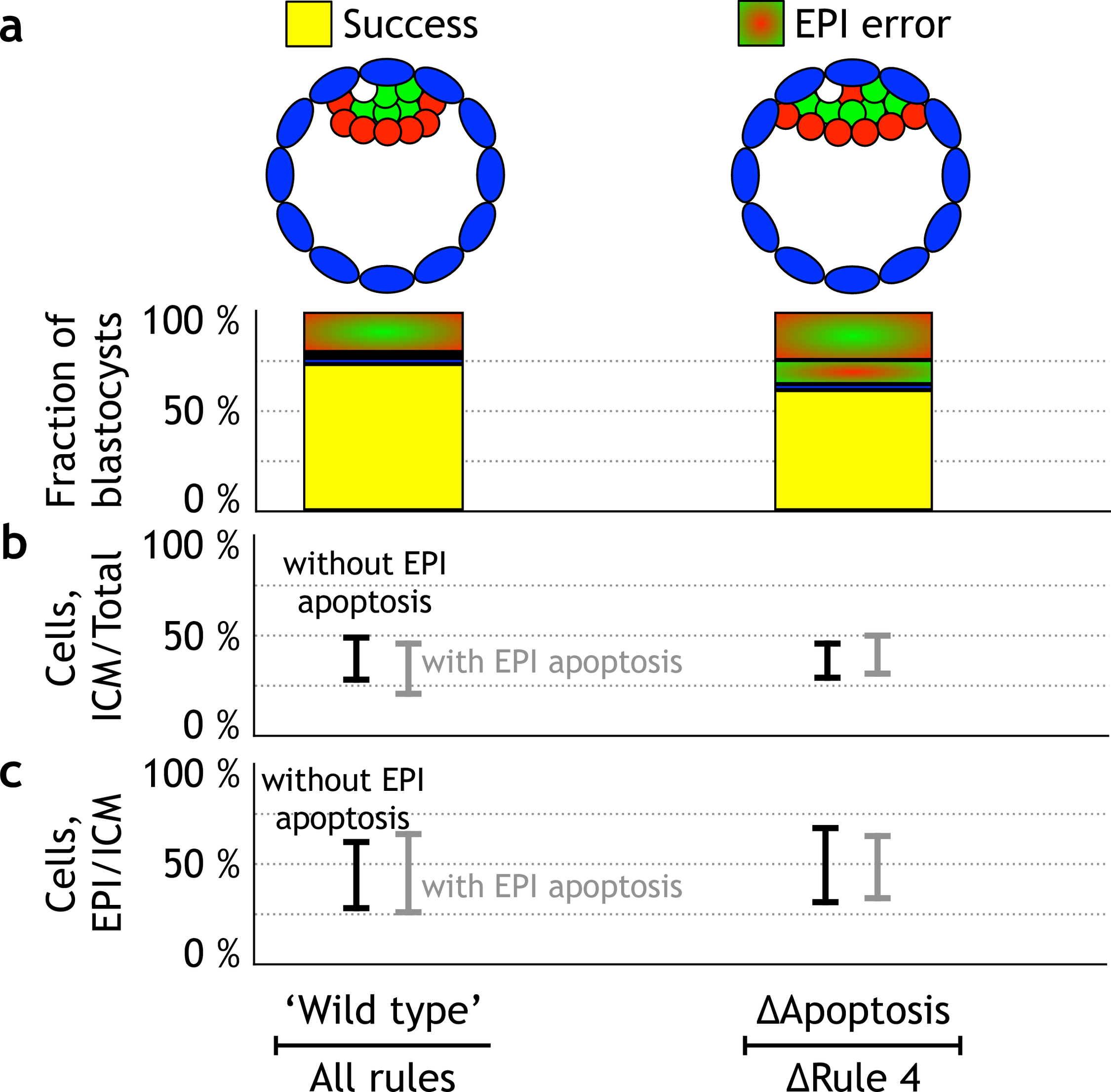

Supplement: S5 Fig — Results of simulations where in addition to our Rule 4, 20% of EPI cells undergo apoptosis at E4.5. This modification results in no significance difference with the earlier results when only misplaced PrE cells undergo apoptosis (compare with Fig 3). The data used to generate the figure is in S2 Data. (TIF) [file pbio.2000737.s005.tif]

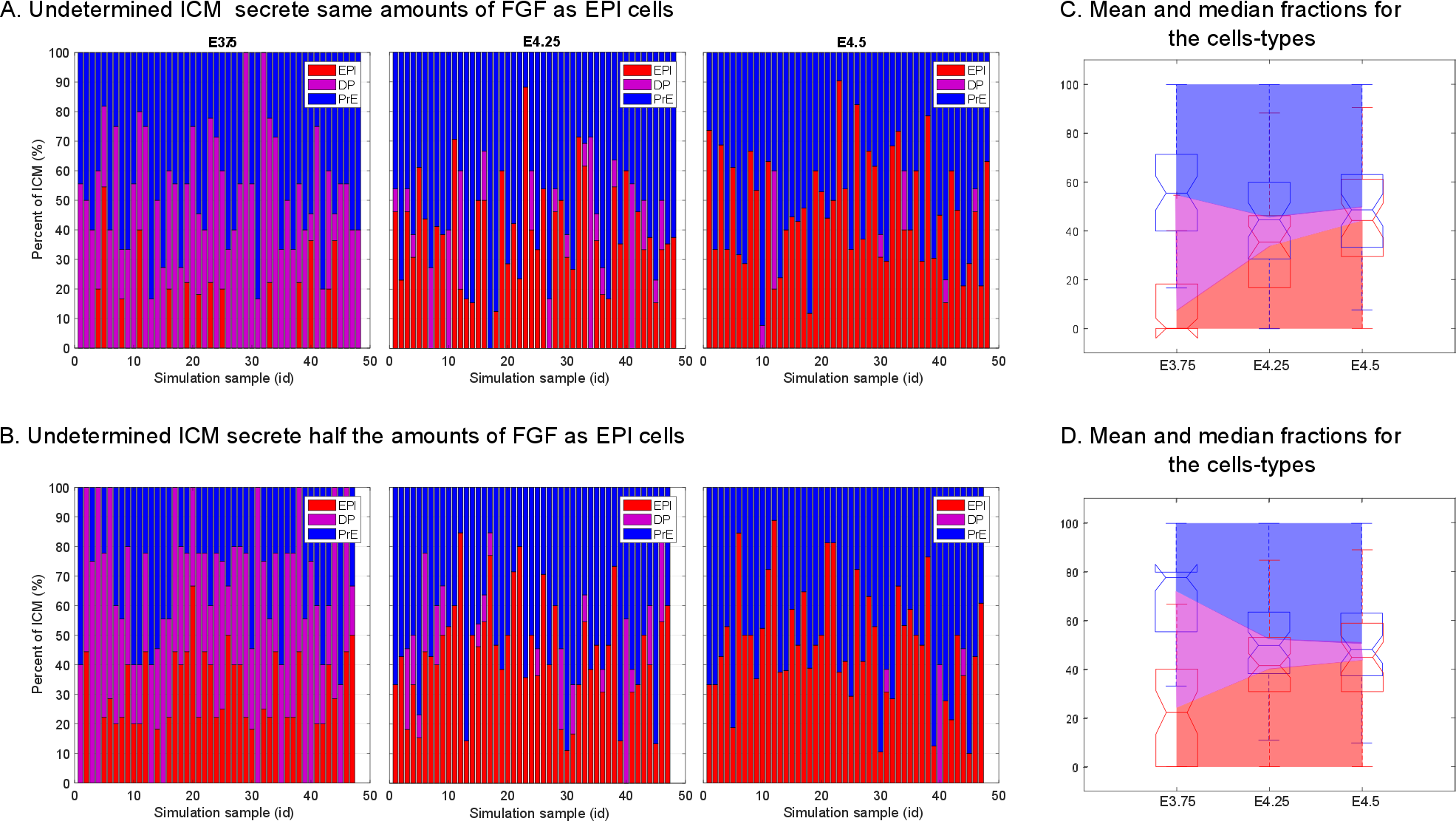

Supplement: S6 Fig — A. Results of the model described in the main text, where undetermined ICM and EPI are assumed to contribute equal amounts of FGF4. B. Results of simulations where undetermined ICM’s contribute half as much FGF4 as EPI. In these simulations, we observed alterations in the initial specification of Epi and PrE at E3.75. Embryos with EPI, but no PrE (in addition to undetermined ICM) appear only in B, but the results at E4.5 are the same in both cases, with ratios converging to 50%. C. and D. The means (boundaries of shaded regions) and medians (notches of the box-plots) of the fraction of cell types are shown for simulations shown in A. and B. The data used to generate this figure is in S3 Data. (TIF) [file pbio.2000737.s006.tif]
